# Resveratrol Preconditioning Protects Against Ischemia-Induced Synaptic Dysfunction and Cofilin Hyperactivation in the Mouse Hippocampal Slice

**DOI:** 10.1007/s13311-023-01386-0

**Published:** 2023-05-19

**Authors:** Iris Escobar, Jing Xu, Charles W. Jackson, Samuel D. Stegelmann, Eric A. Fagerli, Kunjan R. Dave, Miguel A. Perez-Pinzon

**Affiliations:** 1 Peritz Scheinberg Cerebral Vascular Disease Research Laboratories, University of Miami Leonard M. Miller School of Medicine, PO Box 016960, Miami, FL 33101 USA; 2 Department of Neurology, University of Miami Leonard M. Miller School of Medicine, PO Box 016960, Miami, FL 33101 USA; 3 Neuroscience Program, University of Miami Leonard M. Miller School of Medicine, PO Box 016960, Miami, FL 33101 USA

**Keywords:** Cerebral ischemia, Resveratrol preconditioning, Hippocampus, Synaptic dysfunction, Long-term potentiation, Cofilin

## Abstract

Perturbations in synaptic function are major determinants of several neurological diseases and have been associated with cognitive impairments after cerebral ischemia (CI). Although the mechanisms underlying CI-induced synaptic dysfunction have not been well defined, evidence suggests that early hyperactivation of the actin-binding protein, cofilin, plays a role. Given that synaptic impairments manifest shortly after CI, prophylactic strategies may offer a better approach to prevent/mitigate synaptic damage following an ischemic event. Our laboratory has previously demonstrated that resveratrol preconditioning (RPC) promotes cerebral ischemic tolerance, with many groups highlighting beneficial effects of resveratrol treatment on synaptic and cognitive function in other neurological conditions. Herein, we hypothesized that RPC would mitigate hippocampal synaptic dysfunction and pathological cofilin hyperactivation in an ex vivo model of ischemia. Various electrophysiological parameters and synaptic-related protein expression changes were measured under normal and ischemic conditions utilizing acute hippocampal slices derived from adult male mice treated with resveratrol (10 mg/kg) or vehicle 48 h prior. Remarkably, RPC significantly increased the latency to anoxic depolarization, decreased cytosolic calcium accumulation, prevented aberrant increases in synaptic transmission, and rescued deficits in long-term potentiation following ischemia. Additionally, RPC upregulated the expression of the activity-regulated cytoskeleton associated protein, Arc, which was partially required for RPC-mediated attenuation of cofilin hyperactivation. Taken together, these findings support a role for RPC in mitigating CI-induced excitotoxicity, synaptic dysfunction, and pathological over-activation of cofilin. Our study provides further insight into mechanisms underlying RPC-mediated neuroprotection against CI and implicates RPC as a promising strategy to preserve synaptic function after ischemia.

**Supplementary Information:**

The online version contains supplementary material available at 10.1007/s13311-023-01386-0.

## Introduction

Cerebral ischemia (CI) is a pathological condition characterized by rapid loss of blood flow to the brain often due to stroke or cardiac arrest. In selectively vulnerable brain regions, such as the hippocampus, CI typically leads to irreversible cellular injury/death as a consequence of impaired ion homeostasis, massive cell depolarization (anoxic depolarization, AD), and excitotoxicity [1]. In the USA alone, approximately 795,000 new/recurrent strokes and more than 356,000 out-of-hospital cardiac arrests are reported each year [2]. Notably, surviving patients often exhibit debilitating cognitive impairments, spanning across multiple domains including attention, memory, language, perceptual motor, and executive functioning [3–5]. Unfortunately, there are currently no treatment interventions available to facilitate cognitive recovery after injury.

While the central mechanisms underlying post-CI cognitive impairments have not been fully elucidated, evidence indicates that synaptic dysfunction plays a major role. It has been well established that synaptic compartments undergo early structural and functional alterations following ischemia-induced excitotoxicity. In fact, synaptic failure—manifested as spine loss, aberrant spine morphology, and impaired synaptic plasticity—is evident despite the presence of viable neurons after ischemia [6–10]. Early and persistent deficits in synaptic function have been attributed to several mechanisms [11–15], including pathological effects mediated by the actin-binding protein, cofilin [16]. Cofilin, an important cytoskeletal protein involved in modulating actin dynamics, is regulated via its phosphorylation status at serine residue 3 (Ser 3), whereby dephosphorylation promotes its activation. Under conditions of oxidative stress, cofilin hyperactivation and subsequent binding with actin induce the formation of stable cofilin-actin bundles, referred to as “rods,” which have been shown to disrupt normal actin dynamics and synaptic structure, induce synapse loss, block axonal and dendritic transport, and exacerbate mitochondrial membrane potential loss [17–19]. Unsurprisingly, previous studies have demonstrated that exposure to CI induces cofilin hyperactivation and cofilin-actin rod formation [16, 20–22]. To this end, there exists a critical need for the development of therapies targeting early disturbances in synaptic function and their underlying cause after CI.

The utilization of prophylactic strategies, which serve to benefit a large subset of individuals with high proclivity to CI, offers a promising means to combat rapid disturbances in synaptic function given their ability to mitigate deleterious mechanisms of injury at the earliest time possible. Our lab has previously demonstrated that pharmacological preconditioning with the compound resveratrol (3,5,4′-trihydroxystilbene; RSV), herein referred to as resveratrol preconditioning (RPC), promotes ischemic tolerance and renders the brain resistant to subsequent, lethal ischemic insults [23, 24]. RSV is a naturally occurring phytoalexin commonly found in several dietary foods, which has garnered considerable interest as a therapeutic agent against synaptic dysfunction over the years. Across several neurological conditions, studies have demonstrated improvements in hippocampal long-term potentiation (LTP), spine density, and learning/memory following RSV treatment [25–28]. Notably, RSV has been shown to modulate the expression of important synaptic-related proteins, including the activity-regulated cytoskeleton-associated protein (Arc) [29, 30]. Although well known for facilitating activity-dependent endocytosis of AMPARs [31, 32], Arc has also been shown to influence the phosphorylation status of cofilin during activity-dependent states [33]. Previous studies in our laboratory have shown that Arc is required for the protective effects mediated by other forms of pharmacological preconditioning [34]; however, a role for Arc in RPC-induced neuroprotection has yet to be defined. Moreover, RPC-mediated effects on overall synaptic function have not been previously explored in the context of ischemic injury.

In the present study, we aimed to investigate the effects of RPC on early ischemia-induced excitotoxic processes and synaptic dysfunction in the mouse hippocampus. Utilizing ex vivo brain slices, which serve as a suitable model system to study early electrophysiological changes during/after ischemia, we first examined changes in AD onset latency, intracellular calcium accumulation, synaptic transmission, and synaptic plasticity following injury. Additionally, we sought to elucidate potential mechanisms underlying RPC-mediated effects on synaptic function. Given Arc’s protective role in other preconditioning paradigms and its regulatory effect on phospho-cofilin levels, we hypothesized that RPC may protect against synaptic damage during ischemia by upregulating Arc expression and mitigating cofilin hyperactivation. Taken together, our study provides novel evidence supporting the use of RPC as a neuroprotective strategy to combat early CI-induced synaptic dysfunction.

## Materials and Methods

### Animals

All animal usage and experimentation were approved by the Institutional Animal Care and Use Committee at the University of Miami and were in accordance with the US Public Health Service (PHS) Policy on Humane Care and Use of Laboratory Animals and the National Research Council’s Guide for the Care and Use of Laboratory Animals. Euthanasia methods were consistent with the American Veterinary Medical Association (AVMA) guidelines. Male wild-type C57BL/6 J mice were purchased from Jackson Laboratories (Bar Harbor, ME, USA) between 7 and 8 weeks of age. Animals were housed in an AAALAC-accredited facility and maintained on a 12/12-h light/dark cycle at constant temperature and humidity. Mice were given free access to food and water ad libitum. Upon receipt from Jackson Labs, animals were allowed to acclimate for at least 1 week prior to experimental manipulations and numbered for identification by ear punch. Only mice between the ages of 8 and 12 weeks were used for experiments.

### Drug Preparation and Treatments

Trans-resveratrol (Sigma-Aldrich, St Louis, MO, USA) was prepared as previously described [29]. Briefly, resveratrol was dissolved in 100% DMSO at a concentration of 65 mg/mL and aliquots were stored at – 20°C in amber tubes to minimize light exposure. Immediately before use, stock solutions were diluted to a 1 mg/mL working solution (1.5% DMSO) with saline (0.9% NaCl). A single intraperitoneal injection (i.p.) of 10 mg/kg of resveratrol or vehicle (DMSO) was administered to animals approximately 48 h prior to experimental manipulations.

### Acute hippocampal slice preparation

Mice were anesthetized by inhalation of a gas mixture of isoflurane in 30% oxygen (300 mL/min) and 70% nitrous oxide (700 mL/min) delivered by a vaporizer. Mice were then euthanized and their brains were quickly removed with the entire head submerged into ice-cold artificial cerebral spinal fluid (ACSF) bubbled with carbogen (95% O_2_/5% CO_2_). The ASCF solution was prepared by mixing the following components (in mM) in nanopure water: 4.5 KCl, 2 MgSO_4_ • 7H_2_O, 1.25 Na_2_HPO_4_ • 7H_2_O, 126 NaCl, 2 CaCl_2_, 26 NaHCO_3_, 10 glucose (all chemicals from Millipore-Sigma). The ACSF solution was saturated with carbogen and, if necessary, the pH was adjusted to 7.40–7.45 (305–312 mOsm) with HCl. The dissected brain was then placed into an ice-cold slurry of carbogenated ACSF solution and allowed to sit for 1 min. Sagittal slices of 300 μm thickness were sectioned using a Leica VT1000S microtome (Leica Microsystems, Nussloch, Germany) and then transferred into a submerged-type holding chamber containing cold ACSF gassed with carbogen. Slices were gradually brought up to room temperature and allowed to incubate for at least 1.5 h prior to use for experiments. Of note, slices prepared for determination of cytosolic calcium levels were sectioned in sucrose cutting solution containing the following (in mM): 3 KCl, 7 MgCl_2_, 1.25 Na_2_HPO_4_, 60 NaCl, 0.5 CaCl_2_, 28 NaHCO_3_, 5 glucose, 110 sucrose). After sectioning, the hippocampus was dissected out from the slice and placed into a holding chamber containing a 50:50 mixture of sucrose-based ACSF and standard ACSF (composition in mM: 2.5 KCl, 1 MgCl_2_, 1.25 Na_2_HPO_4_, 125 NaCl, 2 CaCl_2_, 25 NaHCO_3_, 10 glucose) for 20 min. Thereafter, slices were transferred to a separate holding chamber containing standard ACSF and allowed to recover for 1.5 h.

### Extracellular Field Recordings

Following the preincubation period, acute slices were transferred into an interface-type recording chamber (Harvard Apparatus, Boston, MA) perfused with carbogenated ACSF (flow rate: 1.5 mL/min) and allowed to acclimate for 20 min prior to recordings. The temperature was maintained at 34°C using an automated temperature controller (Warner Instruments, Holliston, MA) and all relevant equipment were positioned on a vibration isolation table (Technical Manufacturing Co., Peabody, MA) with a surrounding Faraday cage to prevent electrical and mechanical noise. Schaffer collaterals were electrically stimulated with a bipolar tungsten electrode (TST53A05KT; Word Precision Instruments, Sarasota, FL, USA) and stimulus pulses (0.1 ms duration) were generated using a S48 square pulse stimulator equipped with a SIU5 Stimulus Isolation Unit (GRASS Technologies). Evoked field excitatory postsynaptic potentials (fEPSPs) were measured in the stratum radiatum of the CA1 hippocampal subfield with glass microelectrodes filled with 150 mM NaCl (2.5–5 MΩ). Microelectrodes were pulled from borosilicate glass capillaries (1B150-4; World Precision Instruments) with a Sutter P-87 Micropipette Puller (Sutter Instruments, Navato, CA, USA). Signals were amplified using an Axopatch 200B amplifier (Molecular Devices, San Jose, CA, USA)—low-pass filtered at 10 kHz—and digitized at a sampling frequency of 10 or 20 kHz using a Digidata 1200 series interface (Molecular Devices) coupled with Clampex 9 software (PClamp, Molecular Devices). Acquired data were analyzed offline using Clampfit 10.7 software (PClamp, Molecular Devices).

At the beginning of each experiment, input/output (I/O) relationships were determined for each slice. I/O curves were generated by gradually increasing the stimulus strength until the maximal evoked response was reached. The stimulus intensity was adjusted to evoke a fEPSP of about 35–40% of the maximum slope. The negative-going slope of the fEPSP—measured over the 20–80% range between the start of the fEPSP and fEPSP peak amplitude—was used as an index of synaptic strength. In all recordings, a presynaptic fiber volley (FV) preceded the fEPSP; thus, we considered the first point immediately after the fiber volley as the start of the fEPSP. Synaptic transmission was assessed by measuring the relationship between the FV amplitude and fEPSP slope over increasing stimulus intensities obtained from the I/O protocol. The fEPSP slope values were plotted against presynaptic FV amplitudes for each slice. Each set of plotted data was then fit to a linear regression to determine the I/O mean slope for each slice, which was then averaged per group.

To determine changes in paired-pulse facilitation (PPF), a pair of stimulus pulses were delivered to slices over several intra-pulse durations—25, 50, 100, and 150 ms. The paired-pulse ratio (PPR) was measured by dividing the slope value of the fEPSP elicited by the second pulse (S2) by the slope value of the fEPSP elicited by the first pulse (S1). A PPR (S2/S1) value greater than 1 indicated the occurrence of PPF. To assess LTP, baseline fEPSP responses were recorded for 10–30 min, after which LTP was induced using a theta-burst stimulation (TBS) protocol (three trains of stimuli delivered 15 s apart and each train consisting of 10 high-frequency bursts (100 Hz) delivered at 5 Hz) and evoked potentials were recorded (1 stimulus every 30 s) for 50–60 min. Slices that exhibited ≥ 20% baseline variance were excluded from further analysis. Post-TBS values are expressed as the fold change of the average fEPSP slope obtained from baseline recordings. LTP data were analyzed from an average of 11 traces at three selected time intervals (first, middle, and last 5 min of the recording after delivery of TBS). A maximum of two slices were used per animal and each slice was considered an *n* = 1.

### Oxygen and Glucose Deprivation and Anoxic Depolarization

Ischemia was induced ex vivo via oxygen and glucose deprivation (OGD), in which oxygenated ACSF containing glucose was replaced with glucose-free ACSF gassed with 95% N_2_/5% CO_2_. For electrophysiological studies, O_2_ was also replaced with N_2_ in the gaseous phase of the interface-type recording chamber. Acute slices were subjected to OGD until the onset of AD, which was reflected by a large negative direct current (DC) shift in the extracellular field potential. Immediately after AD onset, the medium was switched back to normal oxygenated ACSF; thus, the duration of AD, referring to the time between AD onset and reinstatement of oxygen and glucose, was kept constant at 0 min. We opted to terminate OGD at the onset of AD, rather than use a fixed OGD duration, in order to control for the extent of damage endured by each slice. As the period of time in which OGD persists beyond the onset of AD determines whether synaptic responses recover and cellular injury becomes irreversible [35–37], controlling for the duration of AD offers a better means to maintain similar ischemia-induced changes across slices. After OGD, slices were allowed to recover for 1 h in which evoked fEPSPs were recorded at a rate of 1/30 s.

For both calcium and protein assessments, acute slices were transferred to a homemade submerged-type chamber in which slices rested on cell culture inserts (Millipore-Sigma) or custom-made nylon-mesh inserts. The chamber was placed inside a miniature incubator (Bioscience Tools, Highland, CA, USA) and maintained at 34°C. For induction of ischemia, slices were transferred to wells containing glucose-free ACSF gassed with 95% N_2_/5% CO_2_ for varying durations. In Sham conditions, slices were transferred to a separate chamber containing normal carbogenated ACSF. For protein expression studies, acute slices were harvested immediately following OGD and the hippocampus was dissected out for lysate preparation.

### Intracellular Calcium Measurements

Relative changes in intracellular calcium concentrations were measured spectrophotometrically using a leakage-resistant form of the calcium sensitive fluorescent ratiometric dye, fura-2 AM, known as fura-PE3 AM (Millipore-Sigma). Since fura-PE3 retains nearly identical spectral properties as fura-2 [38], relative calcium concentrations were determined by the ratio of the emission intensity (510 nm) excited by 340 nm and 380 nm measured using a SpectraMax M5 microplate reader (Molecular Devices, Sunnyvale, CA USA). For bulk loading of the dye in acute slices, we adapted a protocol from a previously published study [39]. Fura-PE3 AM was freshly prepared for each experiment, in which 50 µg of the dye was dissolved in 9 µL DMSO and 1 µL Pluronic F-127 (20% solution in DMSO; ThermoFisher Scientific, Rockford, IL, USA) and vortexed thoroughly for at least 10 min to make a 4 mM solution. The stock solution was directly pipetted onto each slice in carbogenated Ca^2+^-free ACSF solution. Application of fura-PE3 in this manner resulted in an initial high concentration of fura-PE3 AM and final concentration of 16 µM in the entire chamber. Slices were loaded in the dark for 50 min at 37°C and washed for 45 min in a separate holding chamber. Following the washout period, a baseline reading was taken. To induce OGD, slices were transferred to a separate submerged chamber and placed onto a custom mesh support. A final reading was taken immediately after exposure to OGD for several durations (5, 10, 15, 20, and 25 min). For a given experiment, 12–14 hippocampal slices were obtained from one animal, which was sufficient to assess cytosolic calcium changes for all OGD durations tested (Sham, 5, 10, 15, 20, and 25 min OGD) in parallel. Two slices from a single animal were pooled together for each OGD duration and was considered an *N* = 1.

### Subcellular Fractionation

Cellular fractions were separated using a protein subcellular fractionation kit (ThermoFisher Scientific) in accordance with the manufacturer’s instructions. Briefly, tissue was gently washed with ice-cold 1 × PBS and homogenized in a pre-chilled glass Dounce with CEB buffer containing protease and phosphatase inhibitors (ThermoFisher Scientific). The homogenate was transferred into a Pierce tissue strainer and centrifuged at 500 × *g* for 5 min (4°C). The supernatant was collected (cytosolic fraction) and the pellet was resuspended in ice-cold MEB buffer containing protease and phosphatase inhibitors. The resuspended pellet was vortexed vigorously for 5 s, incubated for 10 min at 4°C with gentle mixing, and centrifuged at 3000 × *g* for 5 min. The supernatant containing membrane proteins was collected. Pellets were rinsed twice with the appropriate buffer in between steps. Protein concentration was determined using the BioRad DC™ Protein Assay kit (BioRad, Hercules, CA) and 20–50 μg of protein was used for western blot analysis.

### Whole Cell Lysate Preparation

Harvested hippocampal slices were pooled together (4–5 slices), rinsed in ACSF, and homogenized in RIPA buffer containing protease and phosphatase inhibitors (ThermoScientific) with a motor pestle (20 pulses) in an Eppendorf tube. Homogenized samples were incubated for 30 min at 4°C on a rotator and then sonicated twice at low intensity for 8 s. Samples were then centrifuged at 16,000 × *g* for 15 min at 4°C. The supernatant was collected and 30 µg of protein was used for western blot analysis.

### Western Blotting

After protein determination, samples were mixed with 4X Laemmli sample buffer (BioRad, Hercules, CA, USA) containing β-mercaptoethanol and denatured by heating at 95°C for 5–10 min. Western blotting was performed using standard procedures as described in the Supplementary Methods.

### RNA Extraction and qRT-PCR

Total RNA was extracted and purified using the Trizol/RNeasy hybrid or traditional Trizol extraction method. Detailed methods are provided in the Supplementary Methods.

### Antisense Oligodeoxynucleotides

Knockdown of Arc protein expression ex vivo was achieved using antisense oligodeoxynucleotides (AS ODNs). AS ODNs and scrambled control (SCR Ctrl) ODNs were designed as detailed in previous studies [31, 40]. The Arc AS ODN was targeted to a 20-mer sequence of the *Arc* mRNA spanning the translation start site. SCR Ctrl ODNs consisted of randomized nucleotides with the same base composition as the antisense sequence. ODNs were conjugated to cholesterol triethylene glycol (CholTEG) to facilitate cellular uptake (incorporated at the 3′ end of the ODN) and contained phosphorothioate linkages between the three bases on both the 5′ and 3′ ends to confer increased resistance to degradation by endogenous nucleases. Arc AS ODN sequence (asterisks indicate a phosphorothioate bond): 5′-G*T*C*CAGCTCCATCTGGT*C*G*T-CholTEG-3′. SCR Ctrl ODN sequence: 5′-C*G*T*GCACCTCTCGCAGG*T*T*T-CholTEG-3′. ODNs were synthesized by Integrated DNA Technologies (IDT, Coralville, Iowa, IA, USA) and reconstituted in 1X IDTE solution (pH: 8.0; IDT). Upon use, ODNs were diluted in standard ACSF. To validate the efficiency of ODN uptake into cells, an identical Arc AS ODN was designed with the addition of a fluorescein (FAM) tag at the 5′ end.

Following an initial acclimation period at 34°C, acute slices were incubated with either FAM/CholTEG- or CholTEG-conjugated ODNs in a submerged-type chamber for 2 or 6 h at 35°C, respectively. A diluted stock of the AS ODN was directly pipetted onto each slice to give an initial concentration of 250 µM and a final concentration of 5 µM. Slices were then washed in ACSF for 15 min and either fixed for imaging (see slice resectioning below), harvested for protein determinations, or subjected to OGD (see above).

### Slice Resectioning for Fluorescent Imaging

Following incubation with FAM/CholTEG-conjugated ODNs, acute brain slices were fixed in 4% formaldehyde (Pierce, ThermoFisher Scientific, Waltham, MA) in 1 × PBS for 1 h at 4°C on a rocker. Post-fixed slices were washed 3 × 10 min in ice-cold 1 × PBS and then incubated in increasing concentrations of sucrose in 1 × PBS (10%, 20%, and 25%) over the course of 3 days. Slices were flash frozen in liquid nitrogen-cooled isopentane (Sigma-Aldrich) in OCT medium and cut to a thickness of 14 µm using a Leica CM 1850 cryostat (Leica Biosystems, Nussloch, Germany). Coverslips were mounted with Prolong Diamond antifade mountant with DAPI (Invitrogen). Samples were imaged using an inverted Leica Stellaris confocal microscope with × 10 (N.A. 0.40) and × 20 (N.A. 0.75) objectives. Maximum intensity projections were generated from z-stacks (1024 × 1024 pixel resolution, 0.68 µm step size) taken at × 10 and × 40 magnifications (× 1 and × 2 zoom settings, respectively).

### Statistical Analysis

GraphPad Prism 9.4.1 and R-4.1.1 software were used to analyze data. Assumptions of normality were examined using quantile–quantile (Q-Q) plots and the Shapiro–Wilk test. Assumptions of homogeneity of variance were assessed using Levene’s test and the Brown-Forsythe test. For simple comparisons of two sample means, an unpaired two-tailed t-test with Welch’s correction was used. For comparisons of more than two sample means, a one-way analysis of variance (ANOVA) with Dunnett’s (comparing each group to control) or Bonferroni’s multiple comparisons test was used as appropriate. Data with 2 factors were analyzed by two-way ANOVA, followed by Tukey’s HSD or Bonferroni’s post-hoc test as appropriate. LTP data from OGD studies were analyzed by a mixed-model two-way repeated measures (RM) ANOVA (between subject factor: treatment; within-subject factor: time) followed by Bonferroni’s multiple comparisons test. Correlation analyses were assessed with Pearson correlation coefficient. For electrophysiological studies, the experimenter was blinded to the treatment group during the experimental procedure and data collection. Groups were only revealed at the final analysis. Sample sizes are reported under each figure legend, in which “*n*” represents the number of slices per group and “*N*” represents the number of animals per group. All data, except for correlation analyses, are expressed as mean ± SEM. For all statistical analyses, the *p* criterion was 0.05.

## Results

### RPC Protects Against Excitotoxic Mechanisms of Ischemic Injury in Hippocampal Slices

**Fig. 1:**
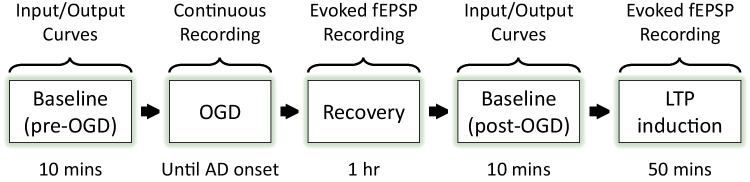
Experimental paradigm for ex vivo electrophysiological recordings in acute hippocampal slices. Input/output curves were generated to determine the stimulus value needed to elicit 35–40% of the maximal evoked synaptic response and to assess synaptic transmission. Changes in short-term synaptic plasticity were assessed using a paired-pulse facilitation (PPF) protocol. Baseline recordings of evoked synaptic responses were obtained for 10 min prior to the induction of ischemia by oxygen and glucose deprivation (OGD). OGD was terminated at the onset of anoxic depolarization (AD) to control for the extent of injury endured by each slice. Thereafter, slices were allowed to recover for 1 h, during which time evoked synaptic responses were recorded. After generating new I/O curves, the paired-pulse response was measured and a new baseline recording was taken. LTP was then induced using a theta-burst stimulation (TBS) protocol and evoked synaptic responses were recorded for 50 min

**Fig. 2:**
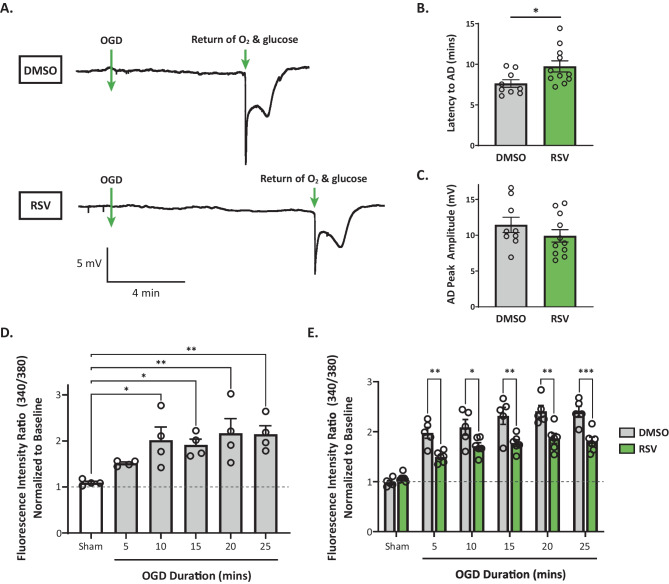
RPC increases latency to AD and reduces cytosolic Ca^2+^ accumulation following OGD in acute hippocampal slices. **A** Example traces of the AD event, characterized by a large negative direct-current shift in the extracellular field potential recording, for each condition. **B**, **C** Quantification of latency to AD (**B**) and peak amplitude (**C**) exhibited by the large AD event. Asterisks indicate significant differences (*p < 0.05) as determined by an unpaired two-tailed t test with Welch’s correction; *n* = 9–11 slices, *N* = 6–7 animals per group. **D**, **E** Relative intracellular calcium levels were measured spectrofluorometrically using a calcium-sensitive fluorescent dye (fura-PE3 AM) in acute hippocampal slices. Calcium influx induced by OGD is represented by an increase in the 340/380 ratio of fluorescence. Values were normalized to pre-OGD (baseline) values indicated by the dashed line. Cytosolic calcium levels were significantly higher following OGD of 10–25 min durations compared to Sham conditions (**D** *N* = 4). Intracellular calcium accumulation was attenuated in RPC-derived slices compared to the control group (**E** *N* = 5–6). Asterisks indicate significant differences (***p < 0.001, **p < 0.01, *p < 0.05) as determined by one-way ANOVA with Dunnet’s post-hoc test (**D**) or two-way ANOVA with Bonferroni’s multiple comparison test (**E**)

To determine the acute effects of RPC on early excitotoxic processes and hippocampal synaptic function following ischemia, we investigated various electrophysiological aspects of RPC-induced ischemic tolerance at the CA3-CA1 synapse by performing extracellular field recordings in acute slices prepared from WT mice preconditioned with RSV or vehicle 48 h prior. The experimental paradigm is outlined in Fig. 1. First, we examined the effects of RPC on AD onset latency, which serves an indicator of ischemic tolerance. As shown in Fig. 2A, B, slices derived from mice preconditioned with RSV exhibited an increased latency to AD compared to control slices (9.74 ± 0.69 vs 7.63 ± 0.47 min; *n* = 9–11 (*N* = 6); *p* = 0.0214). In terms of AD peak amplitude, which reflects the magnitude of the depolarization and the degree of all ion flux changes, no significant differences between groups were observed (Fig. 2C). This finding, however, does not exclude the possibility that RPC may regulate the influx/efflux of specific ions during ischemia.

Interestingly, RSV has previously been shown to modulate intracellular calcium concentrations in different cell types and disease states [41, 42]; thus, it is possible that RPC-mediated neuroprotection involves regulation of cytosolic calcium accumulation during/after ischemia. To explore this possibility, we measured relative changes in intracellular cytosolic calcium concentrations at different OGD durations in acute hippocampal slices using the calcium sensitive fluorescent indicator, fura-PE3 AM. In slices prepared from untreated animals, we observed a moderate increase in relative cytosolic calcium levels after 5 min of OGD, which increased significantly during 10-, 15-, 20-, and 25-min OGD episodes relative to the Sham control (Fig. 2D; *N* = 4; OGD effect: *F*_(5, 18)_ = 4.548; *p* = 0.0074). The steep increase in intracellular Ca^2+^ reached a plateau at 10 min that persisted throughout longer OGD durations. These observations are in agreement with previous studies, which have monitored changes in intracellular calcium in hippocampal slices [43–45].

These experiments were repeated in acute slices derived from mice preconditioned with RSV or vehicle to determine potential effects mediated by RPC. Remarkably, we found a significant treatment effect (*F*_(1, 54)_ = 53.97; *p* < 0.0001) with slices in the RPC group exhibiting attenuated increases in cytosolic Ca^2+^ following OGD compared to the vehicle group (Fig. 2E; *N* = 5–6). Pairwise multiple comparisons revealed that RPC significantly decreased relative intracellular Ca^2+^ levels at all time points analyzed relative to vehicle-derived slices (OGD duration, RSV vs DMSO: 5 min, 1.49 ± 0.05 vs 1.98 ± 0.11; 10 min, 1.71 ± 0.07 vs 2.09 ± 0.16; 15 min, 1.77 ± 0.07 vs 2.32 ± 0.17; 20 min, 1.86 ± 0.10 vs 2.41 ± 0.12; 25 min, 1.80 ± 0.09 vs 2.40 ± 0.11). Additionally, RPC-derived slices plateaued at lower maximal cytosolic Ca^2+^ levels compared to controls. Baseline calcium levels obtained prior to OGD were similar between groups at all OGD durations tested (Supplementary Fig. 1). Taken together, these findings indicate that RPC regulates mechanisms that contribute to AD generation as well as Ca^2+^ homeostasis during ischemia.

### RPC Prevents Aberrant OGD-Induced Increases in Synaptic Transmission

**Fig. 3:**
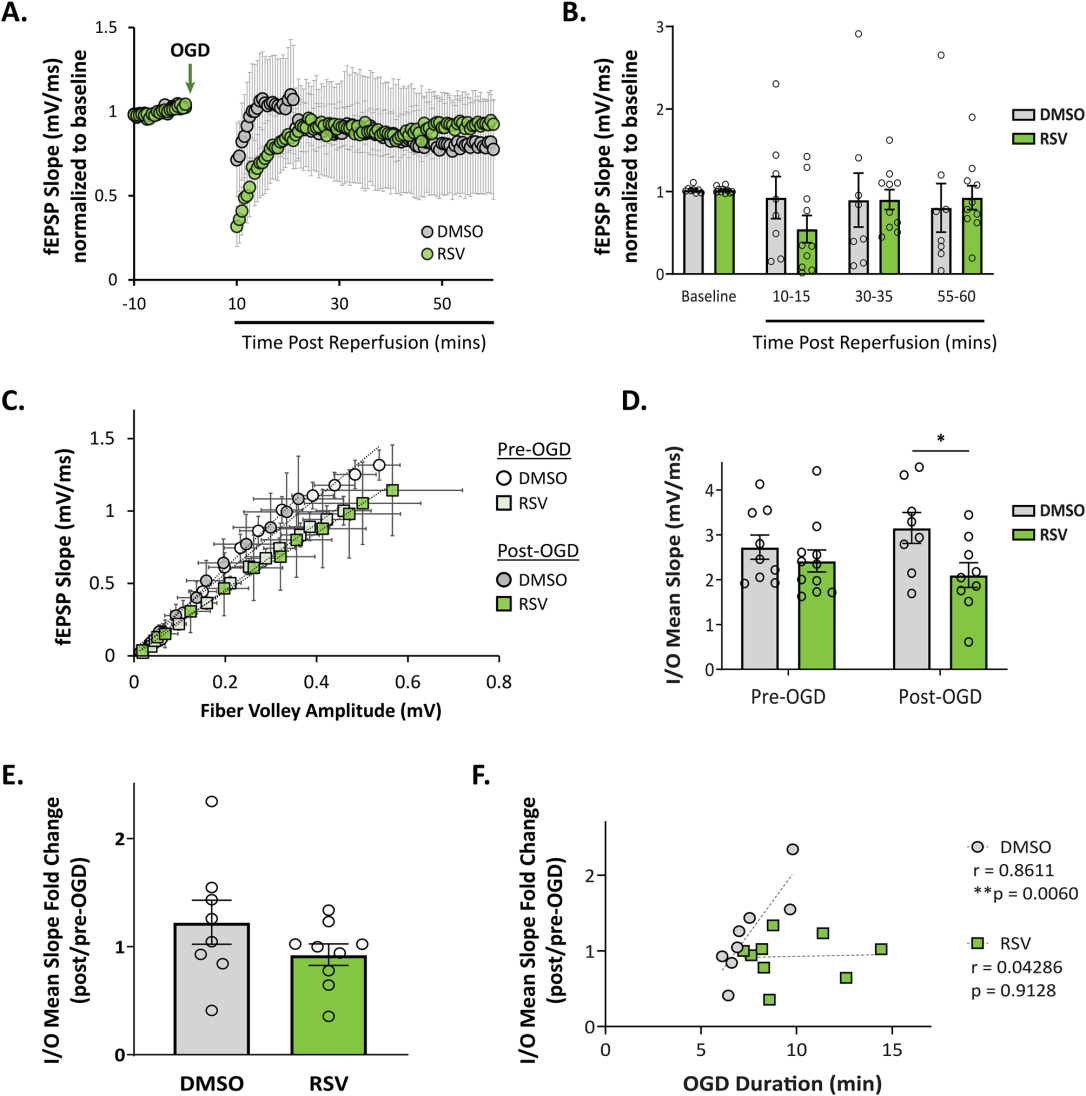
RPC-derived hippocampal slices are resistant to ischemia-induced changes in synaptic transmission. Following AD onset during OGD, slices were reperfused and allowed to recover for 1 h, during which time evoked fEPSPs were recorded. After the recovery period, new stimulus intensities, which elicited 35–40% of the maximal response, were measured.** A**, **B** Averaged time course of fEPSP recovery after OGD in vehicle- and RPC-derived hippocampal slices (*n* = 8–10 slices, *N* = 6 animals per group). The fEPSP slope is expressed as the change of the average baseline response obtained for 10 min before OGD induction. **C**, **F** Input–output (I/O) curve showing the relationship between the pre-synaptic fiber volley (FV) amplitude and fEPSP slope over various stimulus intensities at the CA3-CA1 synapse (**C**). I/O curves were fit to a linear regression for each individual slice per group. The slope of the line generated was averaged for each group shown in the graph (**D**). Asterisks indicate significant differences (*p < 0.05) as determined by two-way ANOVA with Bonferroni’s post-hoc test for multiple comparisons (*p* = 0.0331; *n* = 8–9 slices, *N* = 6 animals per group). The fold change of the I/O mean slope (post-OGD/pre-OGD values) was taken for each slice to assess how much basal synaptic transmission changed before and after OGD (**E**). Pearson’s *r* correlation analyses were used to assess the relationship between the total OGD duration and fold change in basal synaptic transmission before and after OGD (**F**)

Following the same experimental paradigm outlined in Fig. 1, we proceeded to assess the effects of RPC on synaptic function after OGD. As the suppression of synaptic activity is one of the earliest consequences of ischemia [46], we first measured the recovery of evoked fEPSPs. After AD onset, slices were reperfused immediately and continuous recordings were allowed to run for another 10 min, after which synaptic responses were recorded at 30-s intervals for an additional 50 min (total recovery time: 1 h) using the same pre-OGD test stimulus. In both groups, OGD depressed evoked fEPSP responses, which slowly recovered over the course of an hour following reperfusion; there were no significant differences between groups in overall fEPSP recovery (Fig. 3A, B). This is in agreement with previous studies demonstrating that electrophysiological recovery following anoxia is dependent on the duration of AD [47]. Although there was some variability in slice recovery, particularly within the vehicle group, there was no correlation between OGD duration and the fEPSP slope measured 1 h after recovery for both groups (Supplementary Fig. 2A). Notably, despite enduring longer episodes of OGD, the majority of RPC-derived slices (9/10 slices) recovered to near baseline values.

After the recovery period, new stimulus intensities, which elicited 35–40% of the maximal response, were measured to look at changes in synaptic transmission before and after OGD. Synaptic transmission was measured by averaging the mean I/O slope—generated by fitting I/O curve data (fiber volley vs fEPSP slope) to a linear regression—for each slice within a group (Fig. 3C, D). Using the mean I/O slope values, we then calculated the fold change in synaptic transmission within each group before and after OGD (Fig. 3E). Using this measure, we did not observe significant differences between the control and RPC groups. However, levels of synaptic transmission significantly differed between vehicle- and RPC-derived slices when comparing values after OGD (mean I/O slope values: 3.15 ± 0.35 vs 2.10 ± 0.28; Fig. 3C, D), suggesting that ischemia elicited subtle changes sufficient to induce some degree of change between the two groups. Since mice preconditioned with RSV exhibited longer latencies to AD and therefore endured longer durations of OGD compared to controls, we also determined whether there was a relationship between OGD duration and synaptic transmission. We found a positive correlation between the total duration of OGD and I/O mean slope fold change in the control group (Fig. 3F; *r* = 0.8611; *p* = 0.0060), which indicated that longer durations of OGD lead to increased levels of synaptic transmission. This effect, however, was not present in slices derived from RPC-treated mice, suggesting a resistance to ischemia-mediated changes in synaptic transmission.

Given that aberrant increases in synaptic activity likely exacerbate injury in the context of ischemia, we sought to explore potential underlying causes. Interestingly, previous studies have demonstrated that increasing episodes of ischemia induce the targeting of calcium permeable, GluR2-lacking AMPA receptors to synaptic sites [13, 14, 48]. Given that AMPARs mediate the majority of fast excitatory synaptic transmission, we hypothesized that longer episodes of ischemia could induce early changes in AMPAR receptor subunit composition that would favor Ca^2+^ permeability resulting in increased synaptic transmission. To test this hypothesis, cell surface biotinylation experiments were performed within the CA1 region of the hippocampus isolated from acute slices exposed to varying OGD durations. However, we did not observe any changes in surface GluR2 expression, surface/total GluR2 ratios, or surface GluR2/GluR1 ratios following OGD (Supplementary Fig. S3). Therefore, it is likely that changes in synaptic transmission observed 1 h post-OGD may be mediated by other mechanisms.

### RPC Ameliorates OGD-Induced Impairments in Synaptic Plasticity

**Fig. 4:**
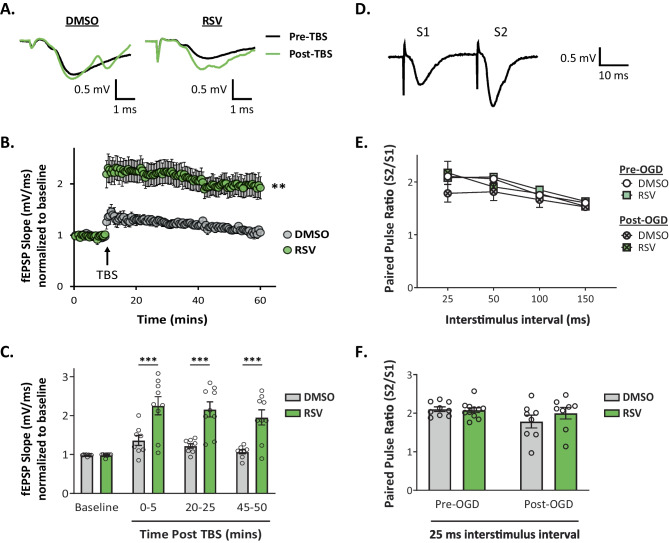
RPC preserves hippocampal LTP after ischemia induced ex vivo. **A** Representative traces of the evoked fEPSP response before (pre-TBS) and 45–50 min after LTP induction (post-TBS) following OGD (average of 11 traces). **B**, **C** Time plot showing mean normalized fEPSP slopes before and after LTP induction in hippocampal slices derived from RPC or vehicle-treated mice (**B**). LTP was induced 1 h following OGD by delivery of three trains of TBS at test stimulus intensity. Each train consisted of 10 bursts (interburst frequency of 5 Hz) and each burst consisted of 4 pulses (intraburst frequency of 100 Hz). Quantitative analysis of the first, middle, and last 5 min of the fEPSP slope following LTP induction (**C**). fEPSP slope averages were taken from eleven traces for each given time bin. Asterisks indicate significant differences (***p < 0.001, **p < 0.01) as determined by 2-way RM ANOVA (mixed model) with post-hoc Bonferroni comparisons; *n* = 8–9 slices, *N* = 6 animals per group. **D** Example trace of the paired-pulse response representing fEPSPs evoked by stimulation pulses delivered with a 25 ms interstimulus interval. The paired pulse ratio (PPR) was calculated as the slope of the second evoked fEPSP (S2) divided by the slope of the first (S1). A ratio above 1 indicates paired-pulse facilitation (PPF). **E** Line graph showing PPR in slices derived from control and RSV-treated mice, before and after OGD. Paired presynaptic fiber stimulation pulses were delivered with varying interpulse intervals ranging from 25 to 150 ms. **F** Average PPR at the 25 ms interstimulus interval. No significant differences were detected in either group before and after OGD; *n* = 8–9 slices, *N* = 6 animals per group

To further evaluate the effects of RPC on synaptic function shortly after OGD, we assessed changes in two well-characterized forms of synaptic plasticity: LTP and paired-pulse facilitation (PPF). While evidence suggests that LTP is impaired in several animal models of both global and focal ischemia as early as 24 h post injury [6, 7, 49–53], few studies have investigated very rapid changes in hippocampal LTP after ischemia. After the 1-h recovery period, LTP was induced by delivery of TBS (refer to Fig. 1). Two-way repeated-measures ANOVA revealed a significant main effect of treatment (Fig. 4A–C; *F*_(1, 15)_ = 15.76; *p* = 0.0012). The slope of the fEPSP at 0–5 min (RSV vs DMSO: 2.25 ± 0.23- vs 1.36 ± 0.13-fold change of baseline slope; *p* = 0.0002) and throughout the 50-min recording period after LTP induction (20–25 min interval, RSV vs DMSO: 2.16 ± 0.20 vs 1.22 ± 0.06; 45–50 min interval, RSV vs DMSO: 1.96 ± 0.20 vs 1.07 ± 0.05) was significantly larger in RPC-derived slices than that of controls (Fig. 4A–C). Although both groups were capable of LTP induction, the magnitude of potentiation was largely reduced in vehicle-derived slices relative to the RPC group, which more closely resembled LTP induction under normal conditions (Supplementary Fig. S4). Moreover, the expression of LTP was significantly impaired in control slices, in which fEPSP slope values returned to near baseline values at the end of the recording period (DMSO, baseline vs 45–50 min interval: 0.98 ± 0.01 vs 1.07 ± 0.05). These findings are consistent with previous studies reporting early impairments in LTP after exposure to anoxia or OGD in hippocampal slices [15, 54] and suggest that RPC preserves LTP shortly after OGD ex vivo. Of note, we found no correlation between total OGD durations and levels of LTP induction (Supplementary Fig. S2B).

Next, we looked at changes in PPF—a form of short-term synaptic plasticity which involves the delivery of two stimuli in rapid succession, separated by a very short interstimulus interval (Fig. 4D). However, we did not observe any differences in either group before and after OGD (Fig. 4E, F), which likely suggests that presynaptic release probability is unaffected acutely following OGD. Altogether, these results indicate that an OGD event terminated at the onset of AD is sufficient to induce synaptic impairments, which can be ameliorated by RPC.

### RPC Modulates the Expression of the Activity-Regulated Cytoskeleton-Associated Protein, Arc

**Fig. 5:**
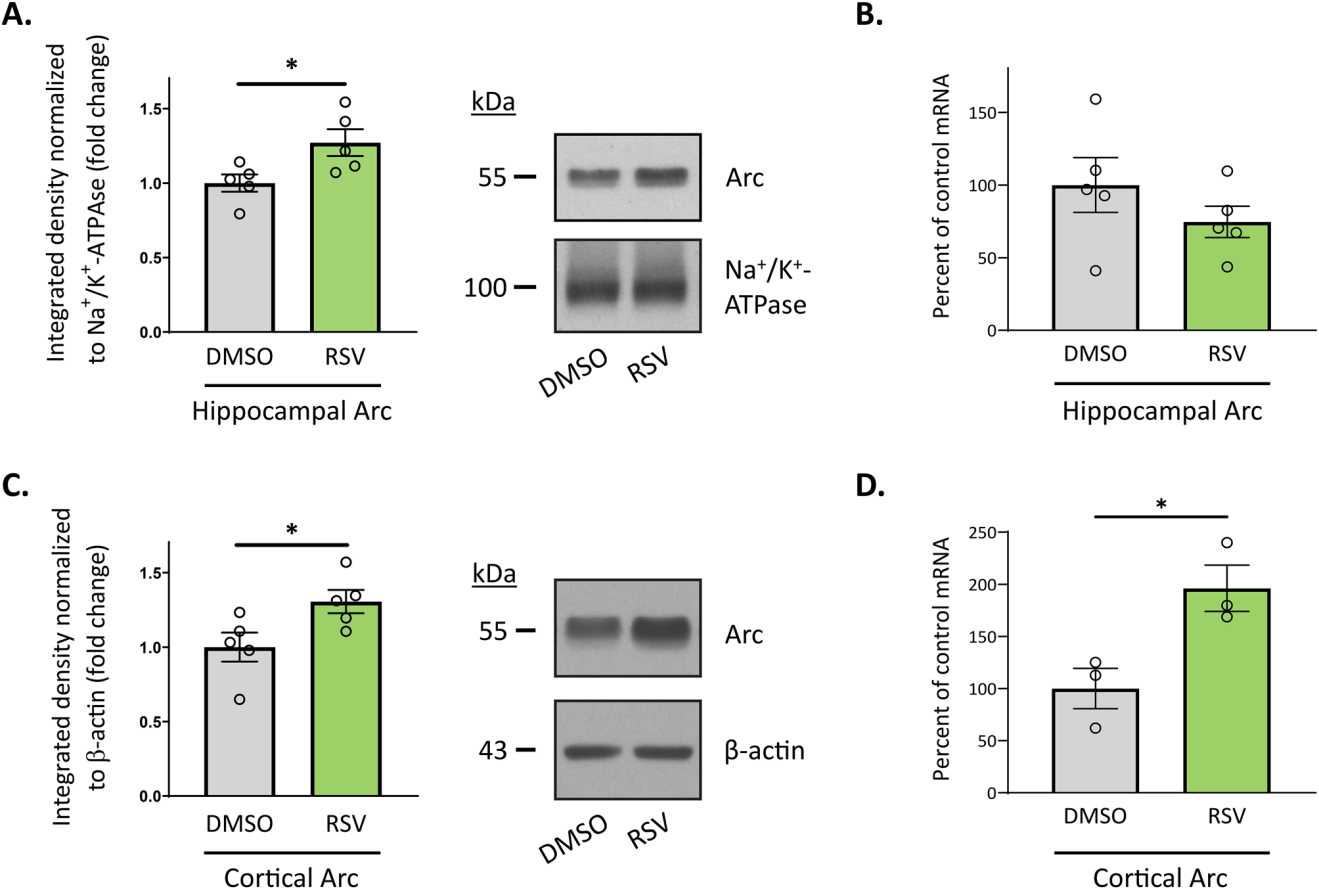
RPC modulates Arc expression in the hippocampus and cortex. Subcellular fractions or RNA was isolated from the hippocampus or cortex of WT mice preconditioned with RSV or vehicle control 48 h prior. **A**, **C** Representative western blots and quantitation of the hippocampal membrane fraction (**A**) or cortical cytosolic fraction (**C**) probed for Arc. Appropriate loading controls were used for each fraction (*N* = 5). **B**, **D** Relative *Arc* mRNA levels, normalized to β-actin, isolated from hippocampal (**B** *N* = 5) or cortical tissue (**D** *N* = 3) as revealed by real-time PCR analysis. Data are expressed as the percentage change compared with control values. Asterisks indicate significant differences (*p < 0.05) as determined by an unpaired, two-tailed t test with Welch’s correction

Alterations in the expression of Arc have been reported at distinct time points after ischemia/reperfusion injury [55–57], which we also find following OGD in primary cortical neurons (Supplementary Fig. S5). Given that different forms of pharmacological preconditioning, including RPC during the extended window of ischemic tolerance, upregulate Arc levels, we hypothesized that induction of delayed ischemic tolerance via RPC also involves the regulation of Arc. To test this, we evaluated cortical and hippocampal Arc protein expression levels within different subcellular compartments using Western blot. We observed a 31% and 27% increase in Arc protein expression 48 h after RPC in the cortex and hippocampus, respectively (Fig. 5A, C). In the cortex, this increase was found in the cytosol (1.00 ± 0.10 vs 1.31 ± 0.08; *p* = 0.0419; *N* = 5) whereas in the hippocampus, Arc protein levels increased in the membrane fraction (1.00 ± 0.06 vs 1.27 ± 0.09; *p* = 0.0396; *N* = 5). To determine whether increases in Arc expression were due to transcriptional activation of the *Arc* gene, we performed qRT-PCR experiments to assess *Arc* mRNA levels following RPC. In the cortex, *Arc* mRNA levels were significantly increased in the RPC group (100 ± 19 vs 196 ± 22; *p* = 0.0316; *N* = 3); however, we did not observe any differences within the hippocampus (Fig. 5B, D).

Since increased levels of hippocampal Arc protein were only found within the membrane fraction of the tissue lysate, we speculated that the total abundance of mRNAs found within the cell might be masking localized effects mediated by RPC. Several signaling pathways regulate Arc expression, whereby *Arc* mRNA is exported to the cytoplasm and further translocated to dendrites to serve local translation at synapses [58]. It is possible that RPC modulates synaptic *Arc* mRNA and protein content. Thus, we measured hippocampal *Arc* mRNA and protein levels in isolated synaptoneurosomes and synaptosomes, respectively, from RPC- and vehicle-treated mice. However, we did not observe significant changes following RPC (Supplementary Fig. 6). It is likely that transcriptional regulation of Arc by RPC is region specific and that other mechanisms may be regulating the protein’s abundance within the hippocampus.

### RPC Ameliorates OGD-Induced Cofilin Hyperactivation

**Fig. 6:**
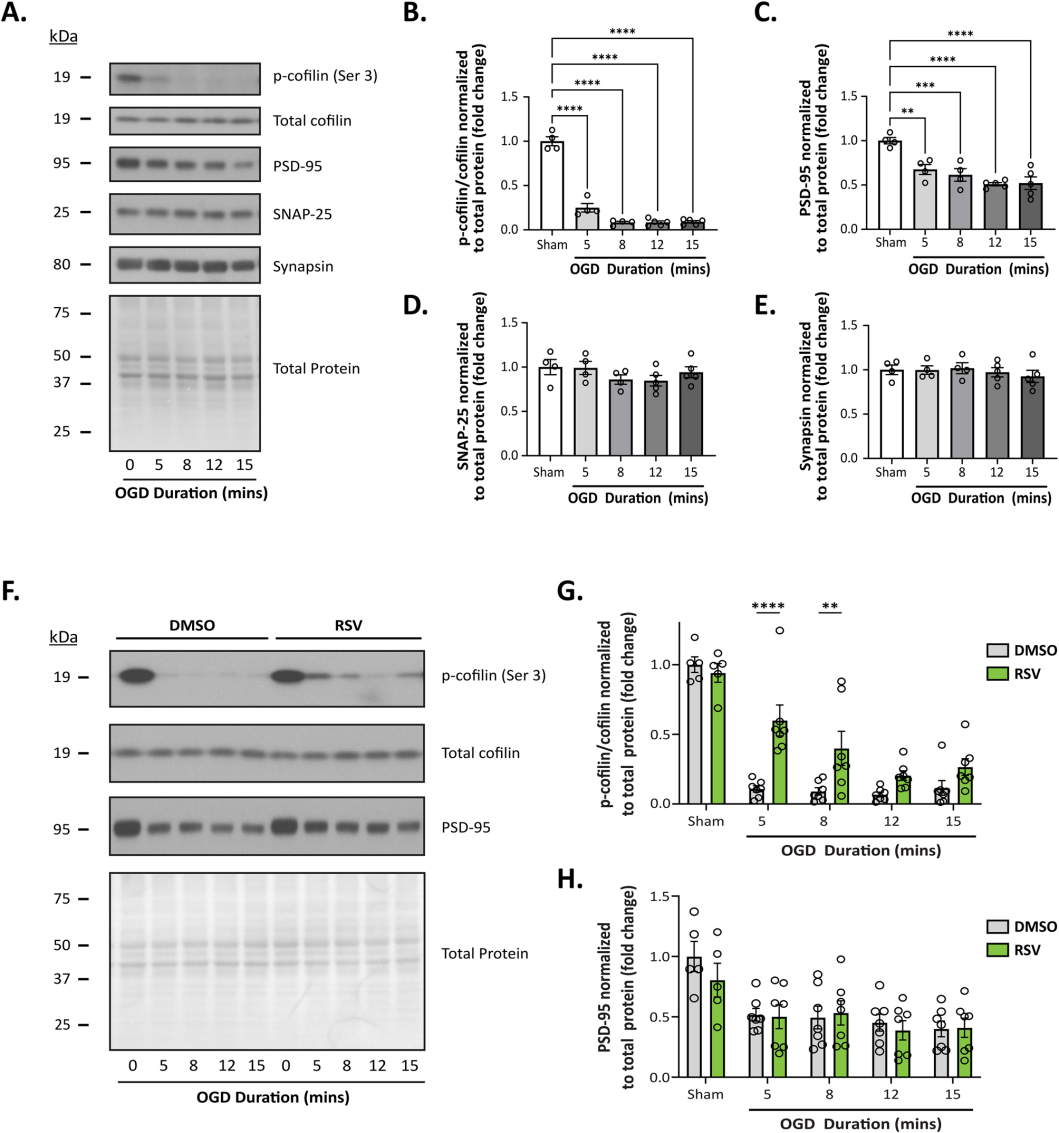
RPC attenuates cofilin hyperactivation following OGD in hippocampal slices. Acute hippocampal slices were prepared from untreated (**A**–**E**) or treated mice injected with either 10 mg/kg RSV or equivalent volume of vehicle solution 48 h prior (**F**–**H**). Slices were exposed to varying durations of OGD (5, 8, 12, and 15 min) and the hippocampus was immediately harvested for whole-cell lysate preparation. Representative western blots (**A** and **F**) and quantitation of protein expression (**B**–**E** and **G**–**H**) corresponding to each blot. Integrated density values for each protein band were normalized to total protein levels—measured by Coomassie blue staining—and the fold change was calculated relative to the control (Sham) group. Asterisks indicate significant differences (****p < 0.0001, ***p < 0.001, **p < 0.01, *p < 0.05) as determined by one-way ANOVA with Dunnet’s post-hoc test (**B**–**E**; *N* = 4–5) or by two-way ANOVA with Bonferroni correction for multiple comparisons (**G**–**H** *N* = 5–7)

Given the observed upregulation of Arc protein following RPC, we sought to define a role for Arc in the RPC-mediated preservation of synaptic function. Aside from Arc’s well-established role in facilitating AMPAR endocytosis, Arc has been shown to influence the phosphorylation status of cofilin, maintaining it in its inactive (phosphorylated) state [33]. Interestingly, previous studies have indicated that ischemia promotes early hyperactivation of cofilin and the subsequent formation of cofilin-actin rods [16, 20–22]. Thus, to determine whether OGD induces similar activation of cofilin in our ex vivo model, we first measured phospho-cofilin levels in acute hippocampal slices subjected to varying durations of OGD (5, 8, 12, and 15 min). Slices were harvested immediately after OGD and whole cell lysates were prepared from the isolated hippocampus. In agreement with previous studies, OGD significantly decreased levels of phospho-cofilin at each OGD duration tested (Fig. 6A, B; OGD effect: *F*_(4, 17)_ = 158.7; *p* < 0.0001; *N* = 4–5). Phospho-cofilin levels were hardly detectable following OGD compared to Sham conditions, indicating that OGD promoted an early and almost complete activation of cofilin.

Since cofilin rod formation has been associated with synaptic loss and concomitant downregulation of PSD-95 expression [59], we also assessed if decreases in phospho-cofilin also corresponded with changes in synaptic markers. Interestingly, we found that ischemia-induced cofilin dephosphorylation was also accompanied by a progressive and marked reduction in PSD-95 expression as the duration of OGD increased (Fig. 6C; *N* = 4–5; OGD effect: *F*_(4, 17)_ = 13.03; *p* < 0.0001). This was expected as several studies have reported the early loss of PSD-95 expression after ischemia [60, 61]. We also looked at the expression of several other synaptic markers including GAP43, neuroligin 1, NMDAR2B, synaptophysin, SNAP-25, synapsin, and SAP102 (Fig. 6 and Supplementary Fig. S7). Interestingly, we only found significant decreases in two other proteins, both of which directly interact with PSD-95: NMDAR2B and neuroligin 1 (Supplementary Fig. 7B, C; OGD effect (NMDAR2B): *F*_(4, 17)_ = 14.58, *p* < 0.0001; OGD effect (neuroligin 1): *F*_(4, 17)_ = 7.158, *p* = 0.0014). Taken together, these findings may explain the observed synaptic deficits in our electrophysiological studies that occur early during the ischemic injury process and suggest a role for cofilin-mediated synaptic deficits following ischemia.

We next questioned whether RPC-mediated protection against CI-induced synaptic dysfunction involves phospho-cofilin regulation. Similar to the aforementioned studies, acute slices were prepared from WT mice preconditioned with RSV or vehicle 48 h prior and subjected to varying durations of OGD (5, 8, 12, and 15 min). Two-way ANOVA revealed a significant main effect of treatment on the expression of phosphorylated cofilin (Fig. 6F–G; *F*_(1, 56)_ = 22.83, *p* < 0.0001; *N* = 5–7). Further analysis showed that phospho-cofilin levels in the RPC group were significantly higher compared to the DMSO group at the 5-min (0.60 ± 0.11 vs 0.11 ± 0.02, *p* < 0.0001) and 8-min OGD durations (0.40 ± 0.12 vs 0.09 ± 0.03, *p* = 0.0074). Phospho-cofilin levels were also higher at OGD exposures of 12 (0.21 ± 0.04 vs 0.70 ± 0.02) and 15 min (0.30 ± 0.10 vs 0.11 ± 0.05); however, these differences were not significant. Given that OGD promoted the loss of other synaptic proteins, we also investigated whether preconditioning with RSV would prevent/reduce such changes following ischemia. However, RPC did not significantly alter PSD-95 levels compared to the DMSO group (Fig. 6H). Likewise, RPC did not alter the expression levels of neuroligin 1 or the NMDAR2B subunit, which were also reduced immediately following ischemia (Supplementary Fig. 7G–I). Overall, these findings indicate that RPC attenuates OGD-induced dephosphorylation (hyperactivation) of cofilin, which has important implications for preventing/reducing the formation of pathological cofilin-actin rod structures after ischemia.

### RPC-Mediated Reductions in Ischemia-Induced Cofilin Hyperactivation Are Partially Arc Dependent

**Fig. 7:**
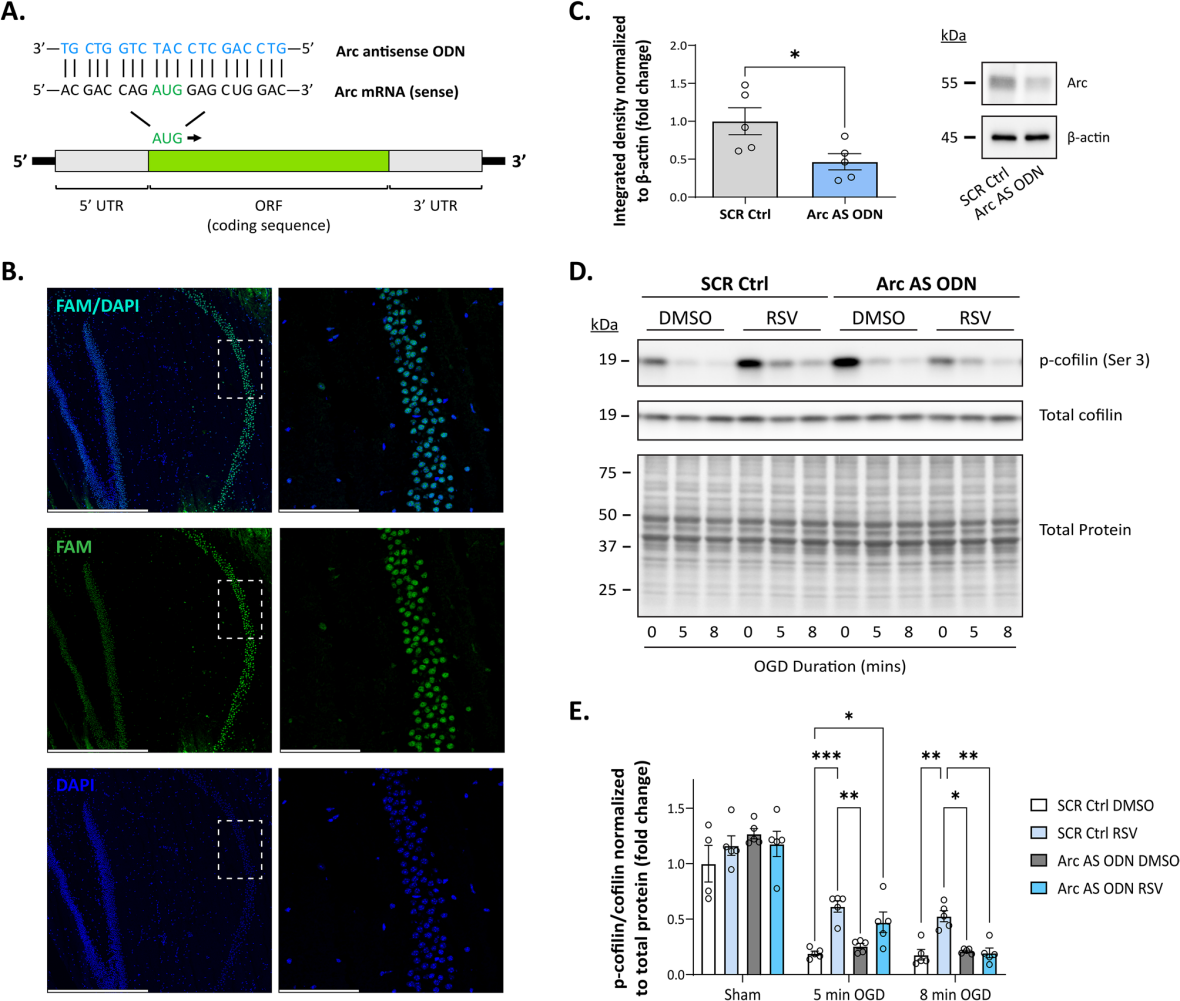
RPC-mediated attenuation of cofilin hyperactivation requires Arc. **A** Schematic showing target location of the Arc AS ODN along the mouse *Arc* mRNA transcript. **B** Fluorescence imaging of resectioned acute hippocampal slices treated with 5 µM Arc AS ODNs conjugated to fluorescein (FAM) and cholesterol triethylene glycol (CholTEG) for 2 h at 35°C, followed by a 15-min washout period. Nuclei were stained with DAPI (blue). Low magnification (left panel, × 10) and high magnification (right panel, × 40) images demonstrate successful internalization of fluorescently labeled ODNs as indicated by co-localization of FAM and DAPI fluorescence. Dashed white boxes denote the magnified regions; scale bar indicates 500 µm (left) or 100 µm (right). **C** Western blot analysis of Arc protein expression in acute hippocampal slices incubated with 5 µM of CholTEG-conjugated scrambled (SCR Ctrl) or Arc AS ODNs for 6 h at 35°C, followed by a 15-min washout period (*N* = 5). **D**–**E** Following incubation with either SCR Ctrl or Arc AS ODNs, acute hippocampal slices derived from RPC or vehicle-treated mice were exposed to Sham conditions or OGD (5- or 8-min durations) and immediately harvested for protein assessments. Representative western blot (**D**) and quantitation (**E**) of p-cofilin expression for each condition (*N* = 4–5). Integrated density values were normalized to total protein levels and data are represented as the fold change relative to the control (SCR Ctrl DMSO) group. Asterisks indicate significant differences (***p < 0.001, **p < 0.01, *p < 0.05) as determined by an unpaired, two-tailed t test with Welch’s correction (**C**) or by two-way ANOVA with Tukey’s multiple comparisons test (**E**)

Since Arc has been associated with the upregulation of cofilin phosphorylation during activity-dependent states [33], we hypothesized that RPC-mediated reductions in cofilin hyperactivation require Arc. To test this hypothesis, we utilized antisense oligodeoxynucleotides (AS ODNs), which have been successfully used in previous studies to modulate gene/protein expression in acute slices [62, 63]. Thus, to knockdown Arc protein expression ex vivo, we designed an AS ODN sequence complementary to the *Arc* mRNA region spanning the translation start site (refer to Fig. 7A) as described in previous studies [31, 40]. Additionally, AS ODNs were conjugated to CholTEG to facilitate cellular uptake. To confirm that AS ODNs were entering cells, we first examined the localization of FAM-tagged ODNs in acute slices. Incubation with a 5 µm concentration of FAM/CholTEG-conjugated AS ODNs for 2 h was sufficient for cells to internalize the ODNs. In resectioned acute slices, we detected fluorescence of FAM-labeled AS ODNs in cells within all hippocampal regions, which co-localized with DAPI staining (Fig. 7B), indicating that the AS ODNs were penetrating cells and entering nuclei. Previous studies have reported that basal Arc expression levels become reduced 6 h following exposure to AS ODNs [31]. Thus, to validate knockdown efficiency, acute slices were incubated with 5 µm SCR Ctrl or Arc AS ODNs for 6 h, after which the hippocampus was isolated for downstream Western blot analysis. Relative to the SCR Ctrl group, Arc AS ODNs induced a 53% reduction in hippocampal Arc protein levels (Fig. 7C; SCR Ctrl 1.00 ± 0.18 vs Arc AS ODN 0.47 ± 0.11; *p* = 0.0385; *N* = 5).

After confirming efficient knockdown of Arc with the Arc AS ODNs, we proceeded to assess whether RPC-mediated attenuation of ischemia-induced cofilin hyperactivation was dependent on Arc expression. RPC- or vehicle-derived slices were incubated with either SCR Ctrl or Arc AS ODNs as described above and then subjected to OGD (5 or 8 min duration) or Sham conditions. As expected, incubation with SCR Ctrl ODNs had no effect on RPC’s ability to attenuate reductions in phospho-cofilin levels following OGD at both durations tested (Fig. 7D–E; 5 min OGD: SCR Ctrl DMSO 0.19 ± 0.02 vs SCR Ctrl RSV 0.61 ± 0.05, *p* = 0.0006; 8 min: SCR Ctrl DMSO 0.18 ± 0.05 vs SCR Ctrl RSV 0.53 ± 0.05, *p* = 0.0056; *N* = 5). Unexpectedly, in the RPC group, phospho-cofilin levels were similar between SCR Ctrl and Arc AS ODN-treated slices (0.47 ± 0.09) following exposure to 5 min of OGD. This was not the case following 8 min of OGD, in which phospho-cofilin levels measured from RPC-derived slices treated with Arc AS ODNs significantly differed from the SCR Ctrl RSV group (SCR Ctrl RSV 0.53 ± 0.05 vs Arc AS ODN RSV 0.20 ± 0.04, *p* = 0.0090; *N* = 5). These findings suggest that RPC’s effects on ischemia-induced cofilin hyperactivation are partly dependent on Arc, whereby RPC may target other pathways during shorter episodes of ischemia to regulate the phosphorylation status of cofilin. Longer exposures to ischemia may overwhelm primary mechanisms induced by RPC to sustain phospho-cofilin levels, thereby leading to the recruitment of secondary measures involving Arc.

## Discussion

Early and persistent disturbances in synaptic function are thought to contribute largely to cognitive impairments following CI. Thus, the development of therapies that effectively target rapid changes in synaptic integrity and function after ischemic injury is of vital importance. The present study investigated the neuroprotective effects of a prophylactic therapy, known as RPC, against CI-induced synaptic impairments in an ex vivo slice model. We report that RPC protects against ischemia-induced excitotoxicity and synaptic dysfunction in the hippocampus as evidenced by RPC-mediated reductions in cytosolic calcium accumulation, increases in the latency to AD, resistance to hyperexcitability, and improvements in LTP shortly after induction of ischemia. In addition, we demonstrate that RPC is capable of attenuating immediate ischemia-induced hyperactivation of cofilin. This effect was partially dependent on Arc expression and offers a potential mechanism by which RPC protects against synaptic dysfunction. These findings suggest that RPC can prevent early and detrimental effects on synapses provoked by ischemia, which have implications for improving cognitive outcomes following injury.

A characteristic feature of both focal and global ischemia is the anoxic depolarization of neuronal and glial cells, which has been associated with steep rises in extracellular glutamate and intracellular calcium concentrations [64, 65]. AD is a type of spreading depolarization that is thought to be a major determinant of irreversible injury as early studies have shown a negative correlation between the duration of AD and the recovery of the orthodromic population spike in field recordings, but no correlation between the total duration of anoxia and the recovery of synaptic responses [37, 47, 66, 67]. Accordingly, our study was designed to isolate electrophysiological changes in response to the AD event, in which the AD duration was kept constant. While previous studies have investigated synaptic plasticity deficits following fixed periods of ischemia [15, 54], this is the first study, to the best of our knowledge, to evaluate alterations in synaptic function and plasticity shortly after exposure to ischemia and AD onset.

Notably, AD is considered a target for therapeutic intervention, as blocking/delaying AD during ischemia markedly improves cellular recovery [68, 69]. In the current study, we report that RPC was able to extend the latency to AD (Fig. 2A, B), suggesting a role for RPC in modulating processes related to AD generation and ensuing excitotoxicity. This has important implications in the clinical setting, as drugs that block/delay AD also have the potential to inhibit milder AD-like events, known as peri-infarct depolarizations (PIDs), that develop following stroke and contribute to a secondary injury process in the penumbra [70, 71]. Considering that AD occurs minutes following stroke onset within the core, AD itself is not a clinical target for improving outcomes. Rather, recurrent PIDs, which occur over prolonged periods and promote infarct expansion [70, 72], represent a feasible target for therapeutic intervention as there exists a window of opportunity to suppress their activity. Since PIDs are generated in a similar manner as AD, treatments that delay/block AD will also affect PID generation—perhaps even more effectively given that energy stores are not completed depleted in the penumbra, at least initially. Importantly, delaying PIDs likely reduces the number of PIDs triggered in a given period of ischemia; thus, maintaining energy reserves for longer durations and expanding the time window for treatments.

Our lab has previously shown that increased Arc expression induced by preconditioning with the PKCɛ activator, ψɛ-receptor of activated C kinase (ψɛRACK), is necessary for neuroprotection against ischemia and the PKCɛ-mediated delay to AD [34]. This effect was attributed to the internalization of AMPAR GluR2 subunits via Arc, which lead to a shift in AMPAR-mediated currents. In our current study, we observe RPC-mediated upregulation of Arc protein expression particularly within the cell membrane. It is possible that similar effects on AMPAR subunits induced by ψɛRACK-mediated upregulation of Arc expression occur with RPC. However, this is unlikely as we did not find that RPC altered the expression of either AMPAR GluR1 or GluR2 subunits in the hippocampus (data not shown). Alternatively, previous studies have demonstrated that increasing glycolytic energy production or preventing depletion of intracellular ATP via creatine supplementation during ischemia significantly delays AD [37, 73, 74]. Interestingly, our lab has previously shown an enhancement in bioenergetic efficiency following RPC during the long-term extended window of ischemic tolerance, in which basal ATP levels and mitochondrial abundance were increased [29]. As the onset of AD reflects the time needed for ATP concentrations to fall below a certain threshold to maintain ionic pumps [75], higher basal levels of ATP induced by RPC likely contribute to the observed delay to AD.

Effects on basal ATP levels and mitochondria may also explain RPC-mediated effects on calcium regulation during ischemia. RSV is known to activate the histone deacetylase, sirtuin1 (Sirt1), which is required for RPC-mediated neuroprotection against CI [76]. Interestingly, Sirt1 can interact with a crucial mediator of mitochondrial biogenesis known as peroxisome proliferator-activated receptor coactivator-1α (PGC-1α) [77]. Although not directly tested in this study, we speculate that a critical mechanism by which RPC attenuates cytosolic calcium accumulation is by promoting mitochondrial biogenesis via Sirt1 and PGC-1α. Increased mitochondrial abundance would allow for more distributed and enhanced sequestration/buffering of intracellular calcium levels, which would not only result in reduced cytosolic calcium accumulation but also a lower intramitochondrial calcium load. However, it is unlikely that RPC targets a single player involved in Ca^2+^ regulation; rather, we suspect that its effects are pleiotropic and may involve multiple mechanisms resulting in decreased Ca^2+^ influx, increased Ca^2+^ efflux, or both. Interestingly, studies have revealed both direct and indirect RSV-mediated effects on intracellular calcium signaling mechanisms via regulation of voltage-gated calcium channels and calcium ATPases [41]. For example, RSV has been previously shown to upregulate the expression of the sarcoplasmic calcium ATPase, which functions to maintain low cytosolic Ca^2+^ levels by pumping free Ca^2+^ ions into the lumen of the endoplasmic reticulum [78]. Certainly, future studies are warranted to elucidate specific RPC targets as well as to identify the specific cell types that benefit from RPC-mediated reductions in cytosolic calcium during ischemia.

Notably, as the initial sites in which apoptotic-like events develop due to calcium overload [79], synapses endure the earliest consequences of ischemia-induced excitotoxic injury. This includes alterations in synaptic structure, neurotransmission, and synaptic plasticity [6, 15, 54]. Evidently, the amelioration of excitotoxic processes during ischemia has implications for preserving synaptic function/plasticity after CI. In our current study, we found that increasing durations of OGD heightened basal levels of synaptic transmission—an effect that was not present in slices derived from RPC-treated mice (Fig. 3F). While increases in synaptic transmission typically reflect enhanced synaptic efficacy under physiological conditions, excessive excitatory synaptic activation during disease states is thought to be pathological [80, 81]. Earlier studies have reported increases in synaptic efficacy between the CA3-CA1 synapse 5–10 h following induction of CI in vivo, which were later followed by a loss of electrophysiological responses that coincided with pyramidal cell degeneration [82, 83]. Thus, we suspect that enhancements in excitatory synaptic transmission observed acutely following OGD may exacerbate excitotoxicity after reperfusion, thereby incurring additional damage to synapses and contributing to the development of delayed neuronal death. The prevention of aberrant ischemia-induced synaptic hyperexcitability may provide a means by which RPC protects against these deleterious effects. Alterations in synaptic transmission could arise from several mechanisms, including effects on pre/postsynaptic sites or reductions in synaptic inhibition. To elucidate whether this effect was mediated by an augmented postsynaptic response, we investigated potential changes in AMPAR subunit composition within the hippocampal CA1 region (Supplementary Fig. S3) as previous reports observed the “switching” of AMPAR subunits at the cell surface after ischemia [13, 14, 48]. However, as we did not detect any changes in AMPAR subunit composition 1 h post-OGD, we suspect that other mechanisms may be at play. Future studies are required to identify potential targets.

In line with previous studies, we also found significant impairments in a well-established form of long-term synaptic plasticity, known as LTP (Fig. 4A–C). LTP is associated with the strengthening of synapses that leads to long-lasting changes in synaptic efficacy and is thought to underlie specific forms of associative learning and memory. Accordingly, deficits in LTP induced by disease conditions have been attributed to memory loss and cognitive decline [84]. Although several studies have reported LTP deficits in different models of CI [6, 7, 49–53], few studies have investigated very early effects of ischemia on hippocampal LTP [15, 54]. Assessing early changes in synaptic plasticity may provide better insight into the efficacy of therapies seeking to ameliorate synaptic dysfunction and degeneration after ischemic injury. Remarkably, we found that RPC was able to rescue ischemia-induced impairments in LTP induction and maintenance, further demonstrating a protective role against synaptic dysfunction. Given that levels of basal synaptic transmission were elevated in vehicle-derived slices, we speculated whether this effect could interfere with the processes underlying LTP induction. Earlier studies have identified a pathological form of synaptic plasticity induced by ischemia known as ischemic LTP (iLTP) [46], which is characterized by an increase in synaptic efficacy and has been previously shown to occlude physiological LTP in a rodent model of cardiac arrest [85]. While our study did not specifically investigate this phenomenon, the observed enhancements in synaptic transmission after OGD suggest a possible role for iLTP in occluding physiological LTP. However, this does not entirely explain our findings as OGD durations varied and the majority of slices within the vehicle group did not express iLTP behavior after reperfusion, yet still exhibited severe LTP deficits.

Interestingly, we find that ischemia altered the expression of important synaptic-related proteins necessary for maintaining normal synaptic function (Fig. 6 and Supplementary Fig. S7). Specifically, we demonstrated early ischemia-induced decreases in the expression of PSD-95, neuroligin 1, and the NMDAR2B subunit, which agree with observations reported in previous studies [60, 86, 87]. However, as RPC failed to rescue OGD-induced reductions in their expression, we sought to investigate OGD effects on other synaptic-related proteins. As mentioned previously, RPC significantly increased the expression of Arc, which is known to interact with a host of different effector proteins in order to bidirectionally regulate synaptic plasticity. In addition to its interactions with the endocytic machinery to regulate AMPAR endocytosis, Arc has been shown to control spine morphology and structural plasticity via regulation of actin dynamics [32]. Various mechanisms of action have been elucidated, but one interesting finding is that Arc can maintain the actin-binding protein, cofilin, in its inactive (phosphorylated) state during LTP consolidation [33]. Cofilin is a critical regulator of actin dynamics/reorganization and under physiological conditions drives actin assembly or disassembly depending on the concentration of cofilin relative to actin. However, in highly oxidative environments, abnormally high levels of active cofilin may promote the bundling of cofilin‐actin filaments into stable rod-like structures, which form due to the generation of intermolecular disulfide bonds between cofilin molecules [17]. Cofilin-actin rods have been detected in the brains of Alzheimer’s disease patients [88] and hyperactivation of cofilin has been shown to contribute to LTP deficits in Alzheimer’s disease models [89, 90]. Ischemic/anoxic insults also induce rapid hyperactivation of cofilin and subsequent cofilin-actin rod formation, which have been attributed to synaptic dysfunction after injury [16, 20–22]. Likewise, we observed robust dephosphorylation of cofilin immediately after OGD exposure (Fig. 6A, B), indicating its early hyperactivation and suggesting a possible role for cofilin in mediating CI-induced impairments in synaptic plasticity.

Consistent with our hypothesis, RPC ameliorated OGD-induced cofilin dephosphorylation (hyperactivation), which was partially dependent on Arc as incubation with Arc AS ODNs blocked RPC-mediated maintenance of phospho-cofilin levels at longer OGD exposures (Figs. 6F, G and 7). Previous studies have demonstrated that Arc does not coimmunoprecipitate with cofilin, indicating an indirect regulatory effect on cofilin activity [91]. Arc, however, does interact with drebrin A, a major regulator of cytoskeletal dynamics, which competes with cofilin for binding to actin filaments [91]. It is possible that Arc cooperates with drebrin A to modulate cofilin activity indirectly. Alternatively, Arc may interact with signaling pathways that serve to activate/inactivate cofilin. Generally, the phosphorylation of cofilin at Ser 3 is regulated by multiple kinases and phosphatases. In adult neurons, Lim domain kinase 1 (LIMK1) primarily phosphorylates/inactivates cofilin, whereas phosphatase slingshot-1L (SSH1L) and chronophin (CIN) dephosphorylate/activate cofilin as well as LIMK1 [17]. Previous studies have demonstrated that ATP depletion enhances CIN-dependent cofilin dephosphorylation [92] and that ischemia-induced cofilin dephosphorylation is mediated by calcium influx and subsequent calcineurin-dependent activation of SSH1L [20]. Arc could possibly carry out its effects by suppressing phosphatase-activating mechanisms during ischemia; however, future studies are necessary to elucidate any specific interactions with signaling cascades involved in phospho-cofilin regulation.

Notably, as knockdown of Arc was insufficient to block RPC-mediated attenuation of cofilin hyperactivation in slices subjected to 5 min of OGD, we suspect that RPC may target various signaling pathways that regulate cofilin phosphorylation independent of Arc. Given the observed reductions in cytosolic calcium accumulation during ischemia (Fig. 2E), RPC may simply repress calcineurin-dependent activation of SSH1L during the initial stages of injury. Generation of reactive oxygen species (ROS) during ischemia may also contribute to cofilin activation as previous studies have demonstrated ROS-mediated activation of SSH1L and cofilin dephosphorylation [93]. Preconditioning or pretreatment with RSV has previously been shown to upregulate cellular antioxidants, including NAD(P)H-quinone oxidoreductase 1, methionine sulfoxide reductases A, and manganese superoxide dismutase [23, 94, 95]. As such, RPC may limit cofilin hyperactivation by protecting against oxidative stress. On the other hand, studies have shown that Sirt1 can suppress microRNA-134, which becomes increased shortly after ischemia [96] and has been shown to inhibit *Limk1* translation [97, 98]. Thus, RPC could potentially sustain phospho-cofilin levels by modulating LIMK1 expression. It is likely that RPC integrates both kinase-activating and phosphatase-inactivating mechanisms during acute exposure to ischemia. During prolonged states of energy deprivation, RPC may rely on secondary defense mechanisms involving Arc to prevent cofilin hyperactivation and subsequent cofilin rod formation that may contribute to synaptic dysfunction.

While our findings highlight a novel protective role for RPC against CI and reveal possible molecular targets for intervention, our study is not without limitations. First, we acknowledge that our experiments were only performed on male mice and, therefore, do not consider potential sex differences. The initial design of this study excluded females in order to improve feasibility and simplify the experimental paradigm. As different stages of the estrous cycle have been shown to influence outcomes after CI, with protective effects observed during the estrus/proestrus stage when estrogen levels are high [99, 100], female studies require examining RPC-mediated effects at distinct phases of the estrous cycle. This becomes further complicated when also considering the effects of estrogen levels on synaptic plasticity [101, 102]. In light of the promising findings outlined in this work, we plan to conduct similar studies in females at variable stages of the estrous cycle in the future. Second, while changes in Arc expression and cofilin activation offer potential mechanisms by which RPC exerts its protective effects, we do not directly show that upregulation of Arc or attenuation of cofilin hyperactivation accounts for the observed preservation of synaptic function mediated by RPC. Studies incorporating knockdown of Arc in vivo following RPC are required to delineate the role of Arc against ischemia-induced synaptic dysfunction. Additionally, as we only measured p-cofilin levels immediately after ischemia, future studies examining the kinetics of p-cofilin recovery following reperfusion are warranted in order to establish a stronger link between cofilin hyperactivation and synaptic plasticity deficits, which were observed 1 h post reperfusion. Given that reperfusion itself is associated with increased ROS production [103] and the formation of cofilin-actin rods persists in the presence of oxidative stress [104], we expect that cofilin will remain hyperactivated for a prolonged period following ischemia/reperfusion injury. Future studies will seek to evaluate RPC’s effects on cofilin hyperactivation and rod accumulation at later time points post-injury and establish whether RPC-mediated preservation of synaptic function/plasticity requires regulation of cofilin phosphorylation status.

Despite these limitations, the work presented here lends further support to the use of RPC as a viable therapeutic strategy to limit ischemic damage and potentially improve cognitive outcomes after injury. Although it is impossible to predict the occurrence of cerebral ischemic events, RPC can be applied to a variety of clinical scenarios. For example, patients undergoing certain procedures that carry risk of causing CI, such as coronary artery bypass grafting and carotid endarterectomy, could directly benefit from RPC. In addition, our findings hold promise for the potential implementation of chronic preconditioning interventions in high-risk patients, such as those with a history of stroke/transient ischemic attacks or those with defined standard risk factors (i.e., age, genetic disposition, hypertension, diabetes). In such cases, there may be a future where pharmacological IPC mimetics, such as RPC, can be taken for prolonged periods in a similar manner as antiplatelet treatments, which are currently being used for long-term secondary stroke prevention [105]. Notably, the maximal window for neuroprotection following the administration of a single dose of resveratrol in mice is 14 days [106]. We have not tested RPC beyond this interval; however, it is possible that the neuroprotective effects mediated by RPC can persist for longer periods—potentially sustained by a positive-feedback mechanism. Certainly, more research in the translational application of preconditioning is warranted to confirm its effectiveness in humans as well as a more defined therapeutic window that is relevant for individuals with high proclivity to CI.

## Conclusions

In summary, our findings indicate a protective role for RPC in the rapid development of hippocampal synaptic deficits following CI. Using the acute hippocampal slice as a model, we demonstrate an RPC-mediated attenuation of excitotoxic events, manifested as reductions in excessive cytosolic calcium accumulation and increased latency to AD. Consistent with these observations, we also show rescue of ischemia-induced synaptic hyperexcitability and LTP impairments in RPC-derived slices. The ability of RPC to preserve synaptic function was partly attributed to its upregulation of Arc, which was required for sustained RPC-mediated attenuation of cofilin hyperactivation. In view of these observations, it is plausible that RPC prevents the early over-activation of cofilin to mitigate sequential pathological effects on synaptic function. Taken together, our findings underscore the use of RSV as an effective preconditioning agent against CI-induced synaptic dysfunction and add further support to the large body of evidence implicating RSV as a promising therapeutic to combat cognitive decline.

## Supplementary Information

Below is the link to the electronic supplementary material.

Supplementary file1 (PDF 164 kb)

Supplementary file2 (PDF 320 kb)

Supplementary file3 (PDF 1591 kb)

Supplementary file4 (PDF 642 kb)

Supplementary file5 (PDF 249 kb)

Supplementary file6 (PDF 325 kb)

Supplementary file7 (PDF 3121 kb)

Supplementary file8 (PDF 533 kb)

Supplementary file9 (PDF 533 kb)

Supplementary file10 (PDF 533 kb)

Supplementary file11 (PDF 533 kb)

Supplementary file12 (PDF 533 kb)

Supplementary file13 (PDF 541 kb)

Supplementary file14 (PDF 524 kb)

## Data Availability

Data are available for sharing from the corresponding author upon request.
