# Supplementary figures and images for: Resveratrol Preconditioning Protects Against Ischemia-Induced Synaptic Dysfunction and Cofilin Hyperactivation in the Mouse Hippocampal Slice
Source: Neurotherapeutics. 2023 May 19;20(4):1177–97. doi: 10.1007/s13311-023-01386-0 (PMC10457274; doi:10.1007/s13311-023-01386-0)

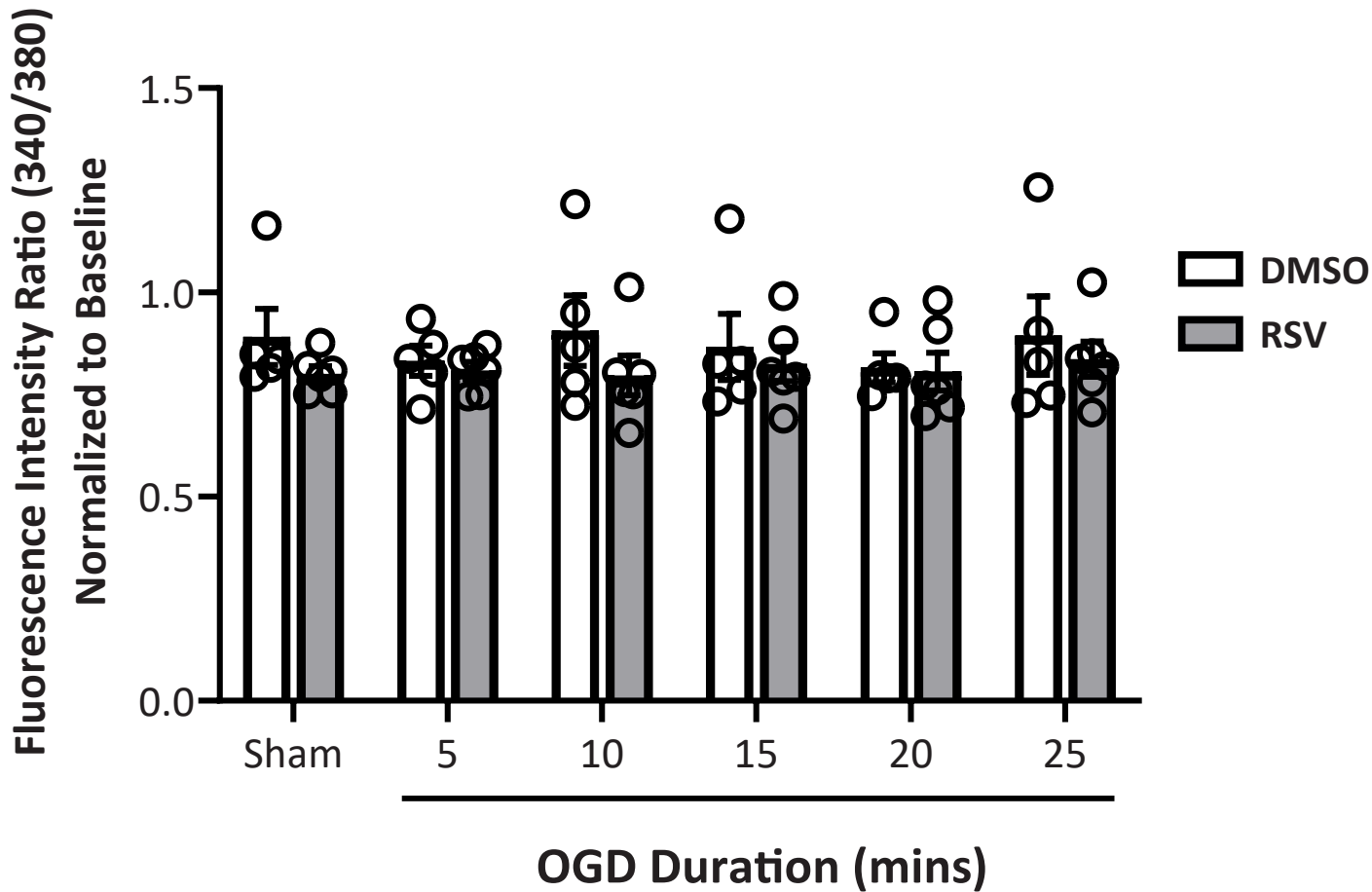

Supplement: Supplementary file 1 — Supplementary file1 (PDF 164 kb) [file 13311_2023_1386_MOESM1_ESM.pdf]

**A.**

fEPSP Slope (mV/ms) post-OGD  
(normalized to baseline)

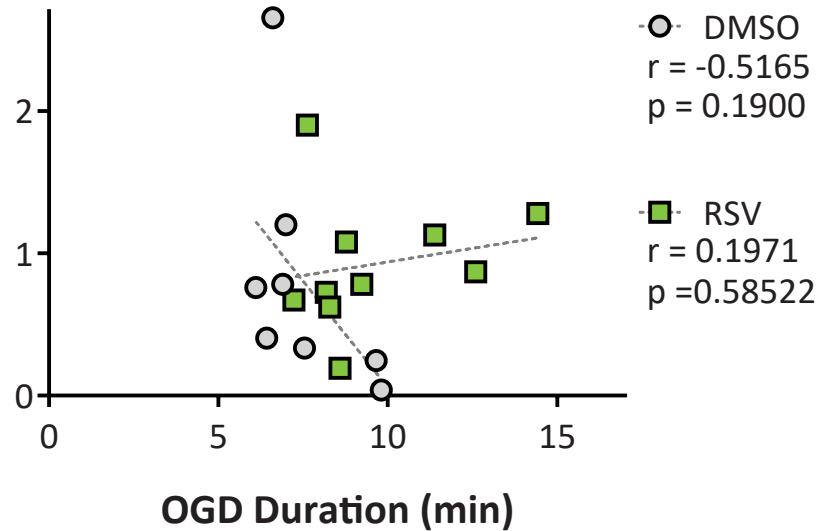**B.**

LTP induction level (mV/ms)

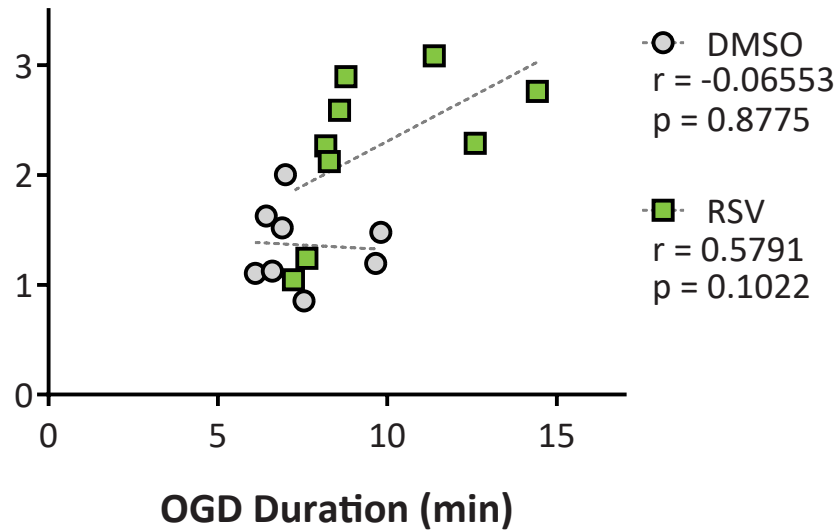

Supplement: Supplementary file 2 — Supplementary file2 (PDF 320 kb) [file 13311_2023_1386_MOESM2_ESM.pdf]

**A.**

Process CA1 for cell  
surface biotinyaltion

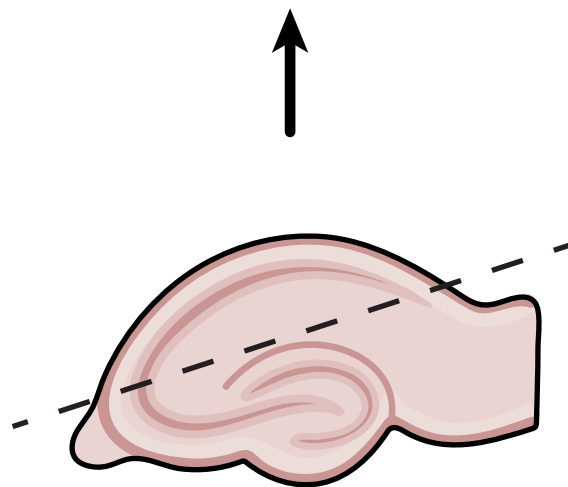**B.**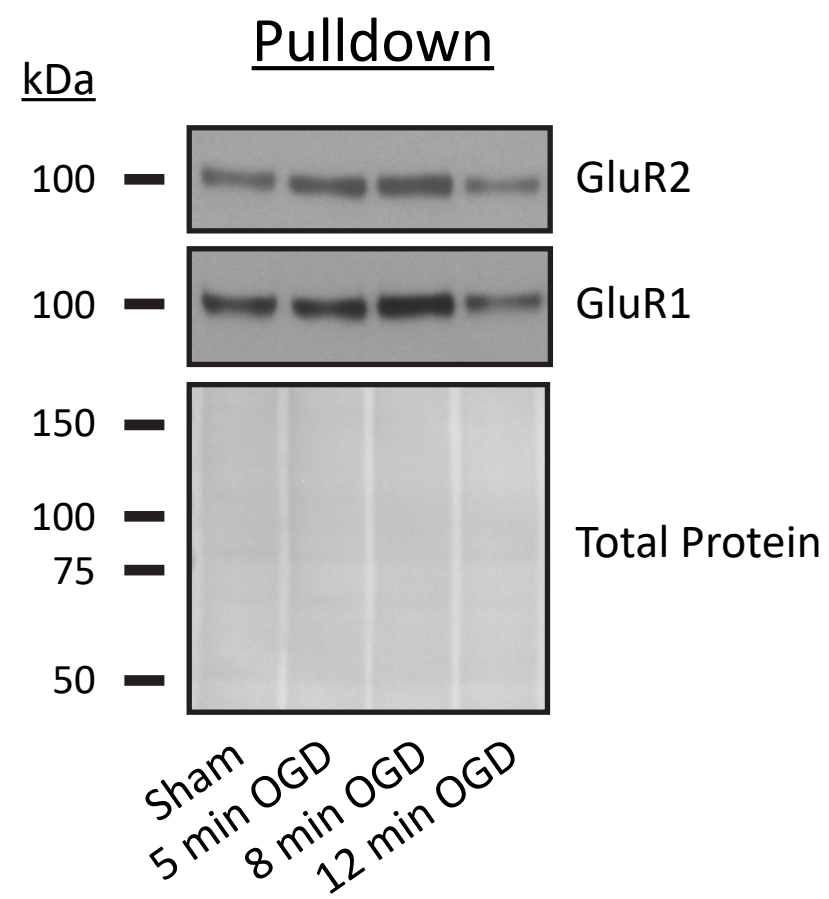**C.**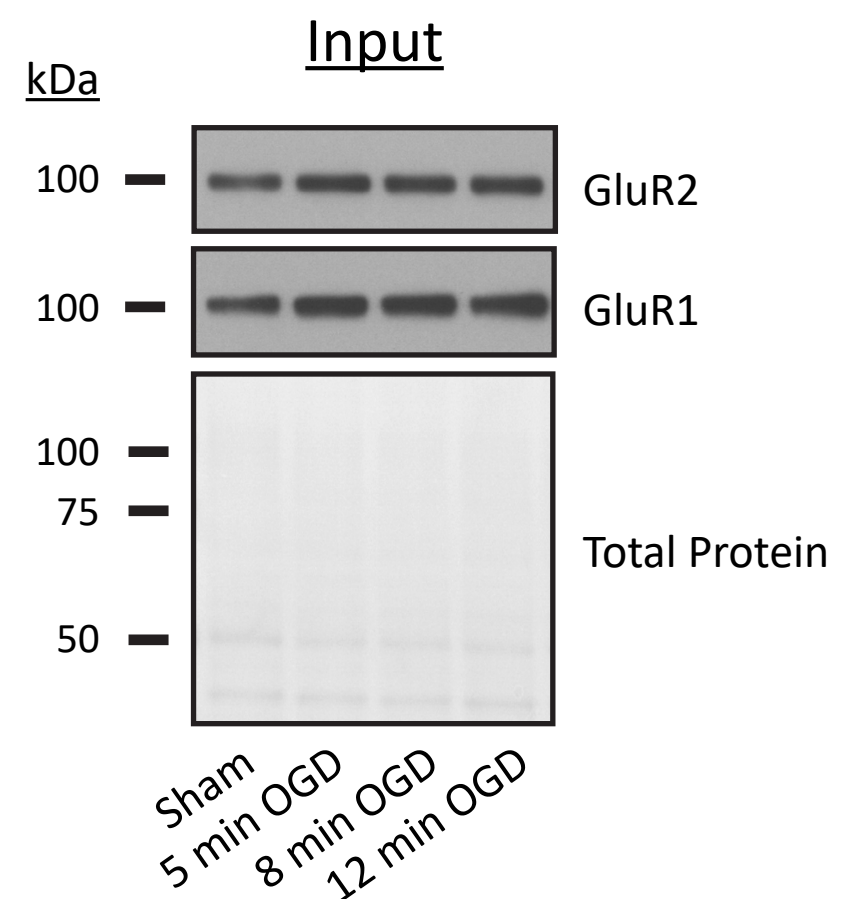**D.**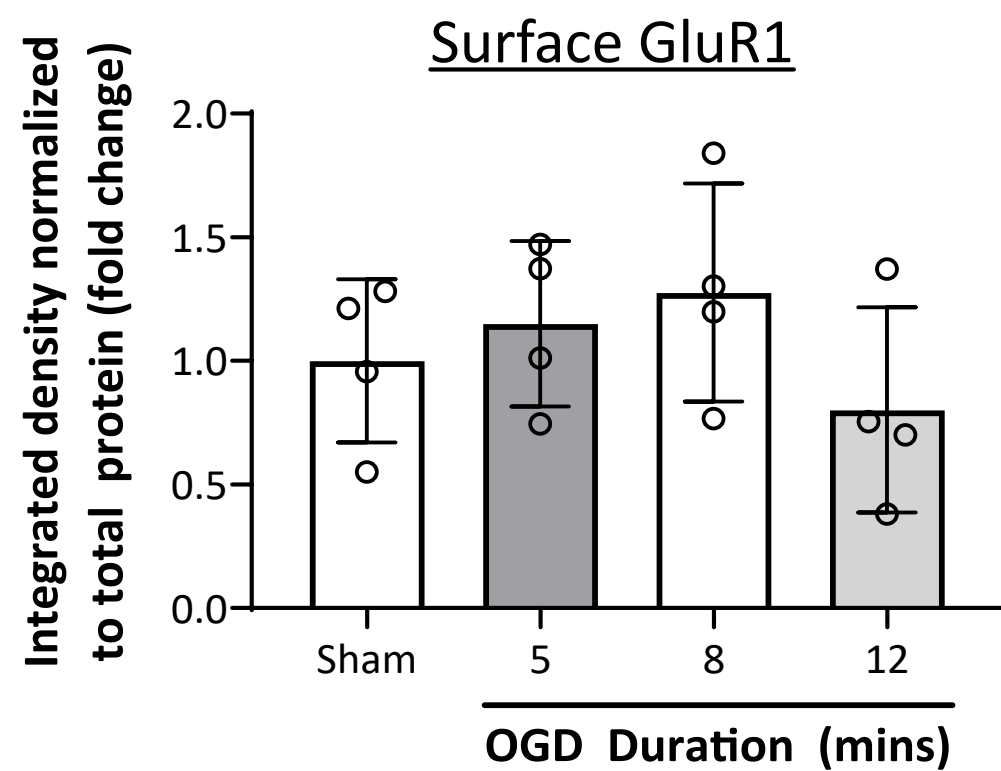**E.**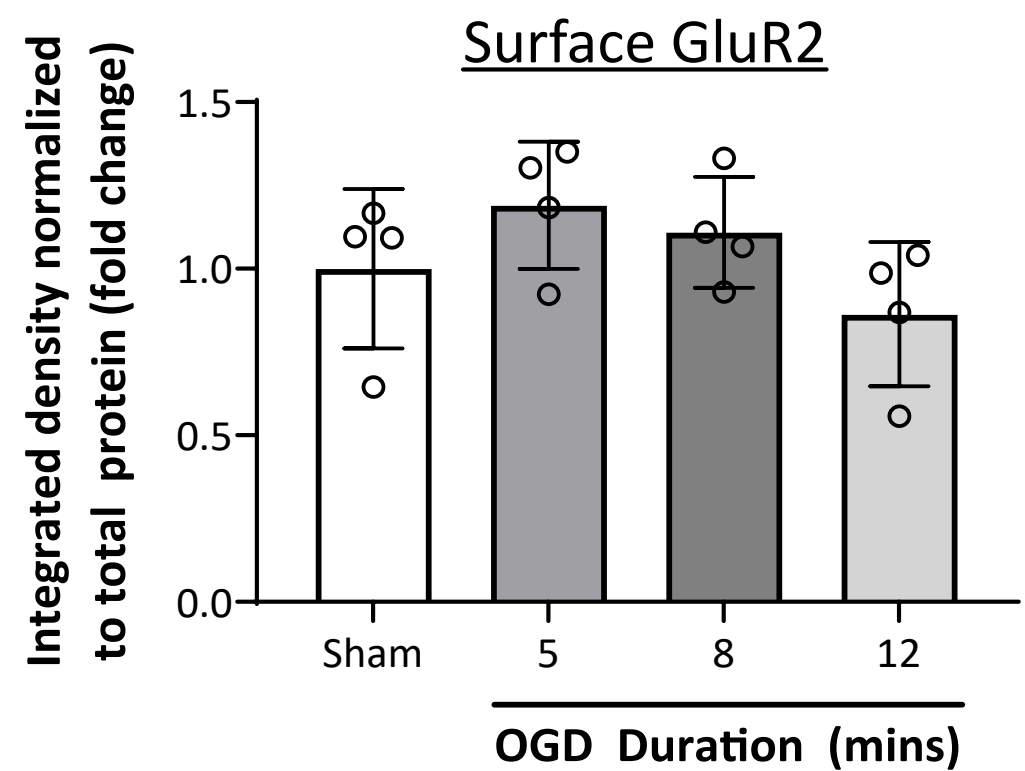**F.**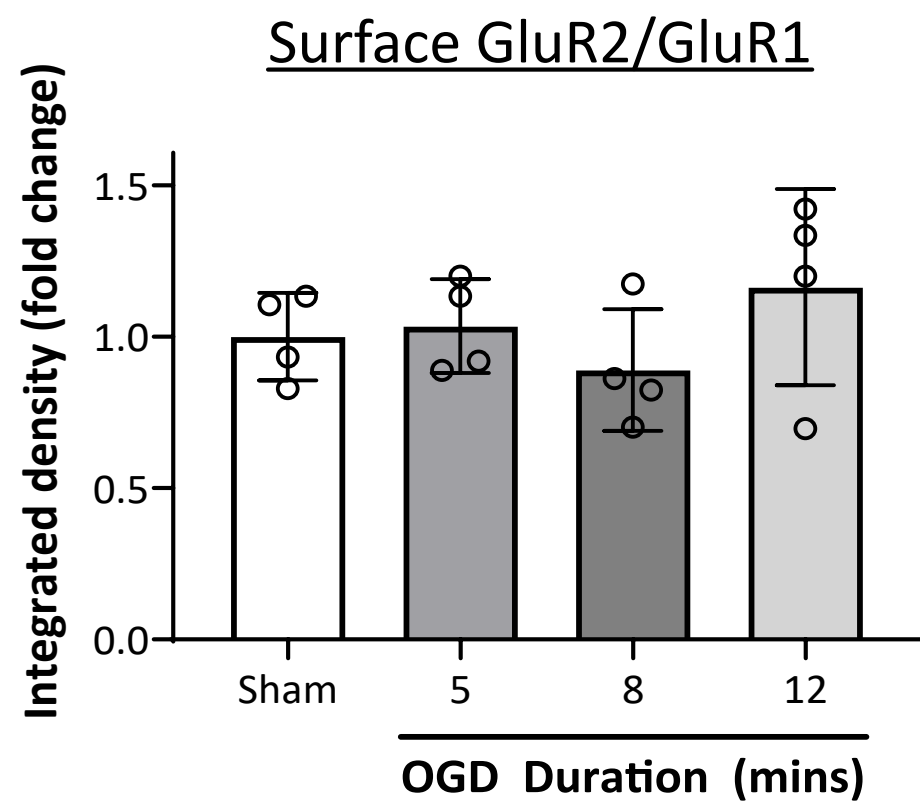**G.**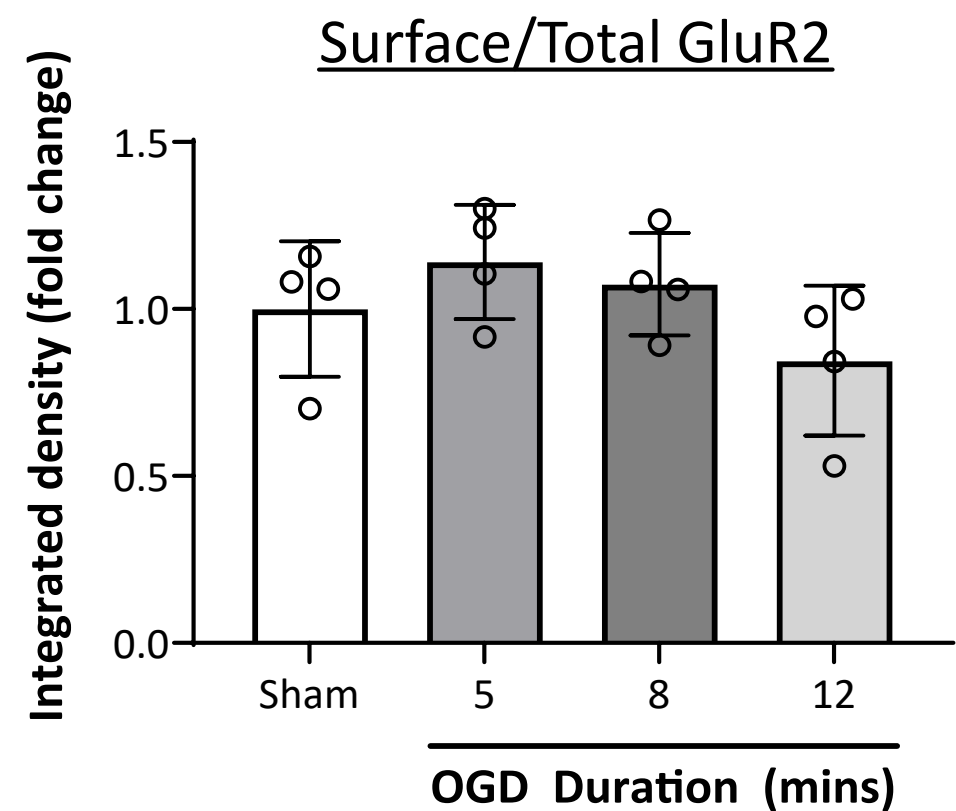

Supplement: Supplementary file 3 — Supplementary file3 (PDF 1591 kb) [file 13311_2023_1386_MOESM3_ESM.pdf]

**A.**

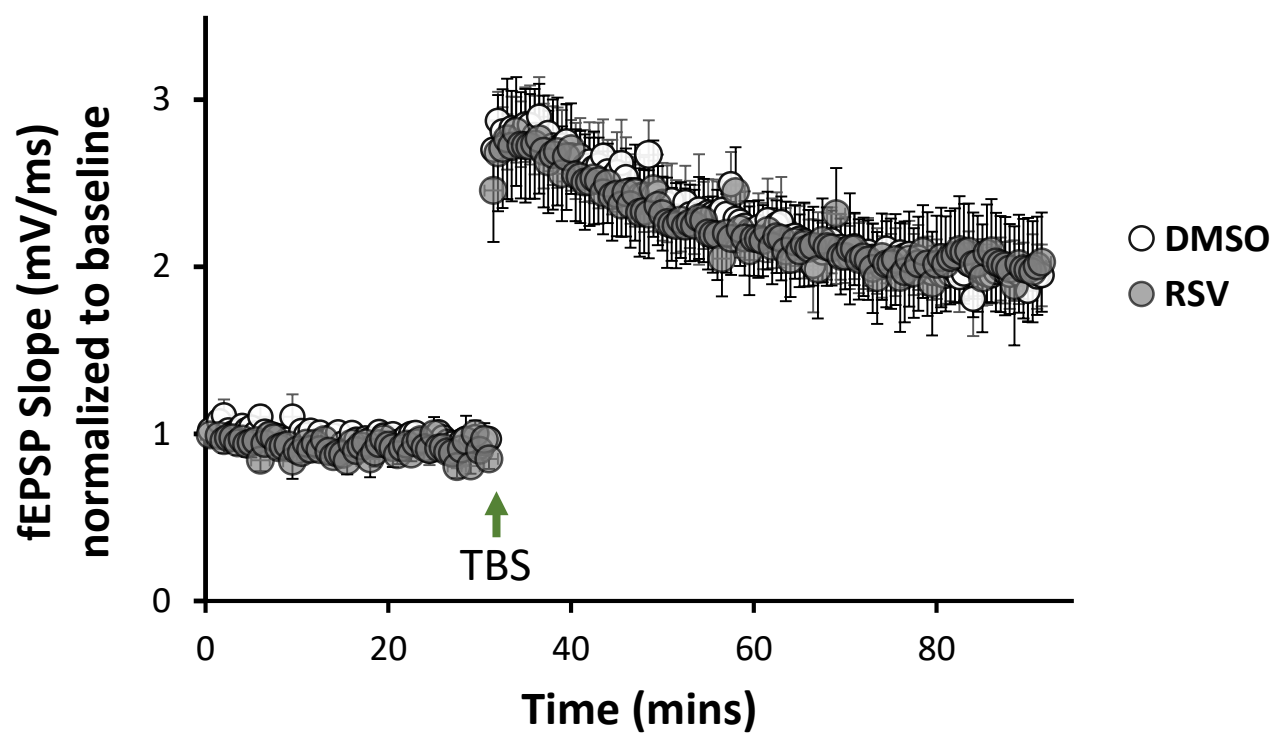

**B.**

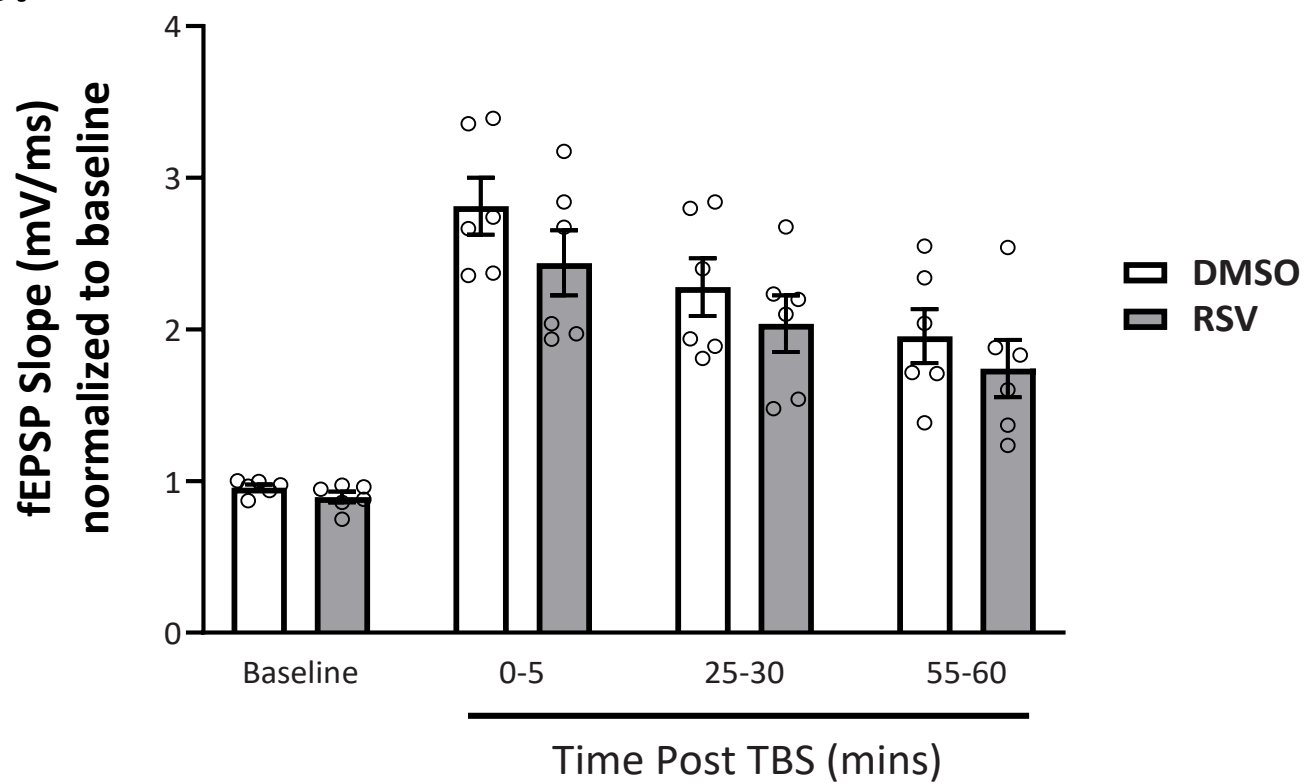

Supplement: Supplementary file 4 — Supplementary file4 (PDF 642 kb) [file 13311_2023_1386_MOESM4_ESM.pdf]

Percent of Sham mRNA

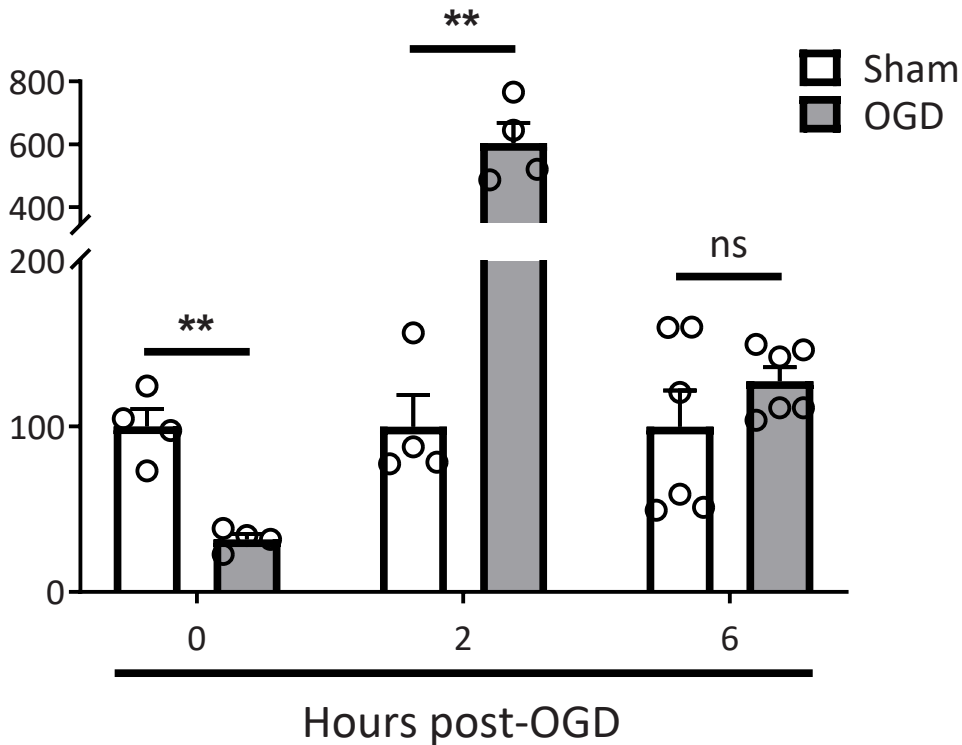

Supplement: Supplementary file 5 — Supplementary file5 (PDF 249 kb) [file 13311_2023_1386_MOESM5_ESM.pdf]

**A.**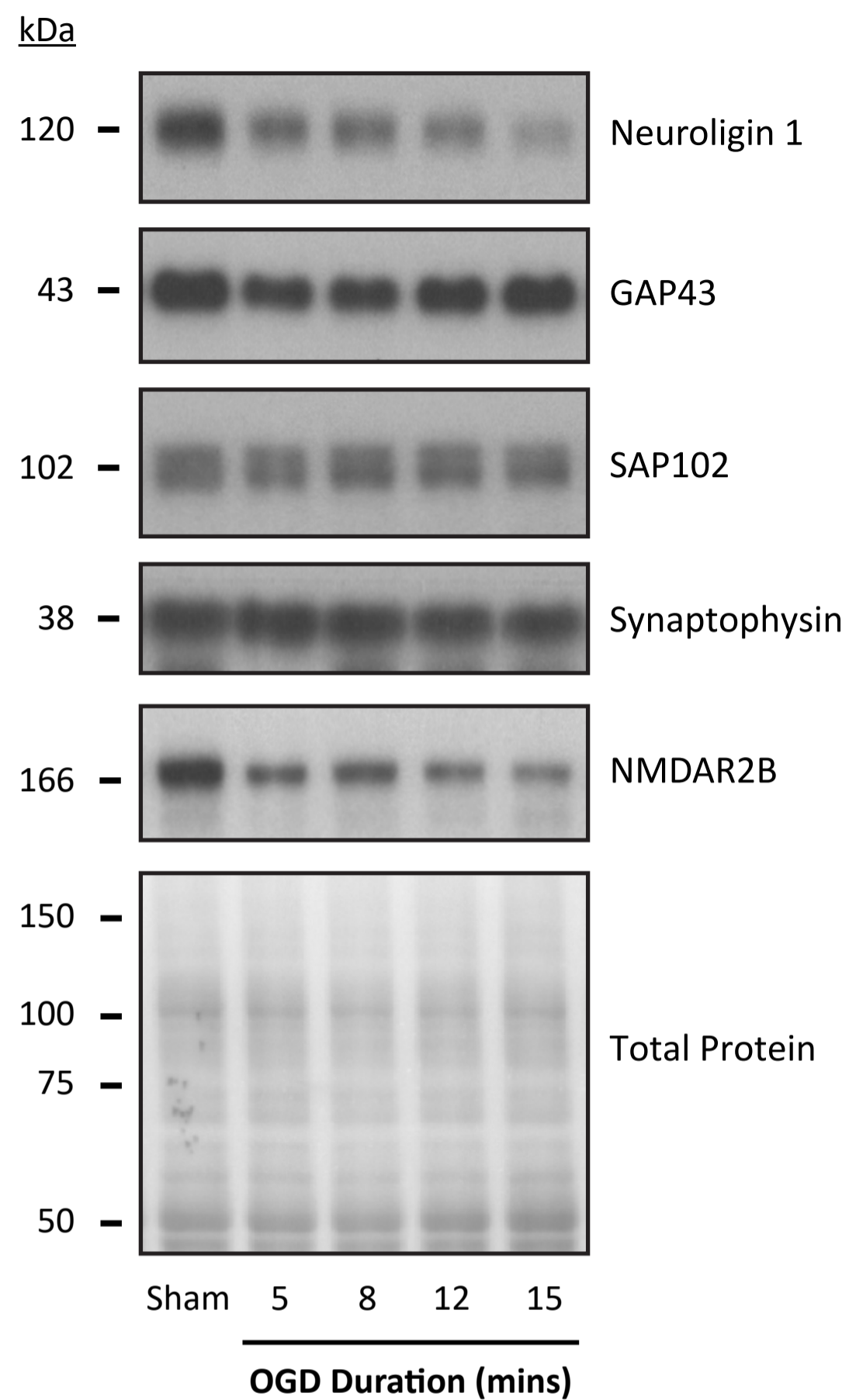**B.**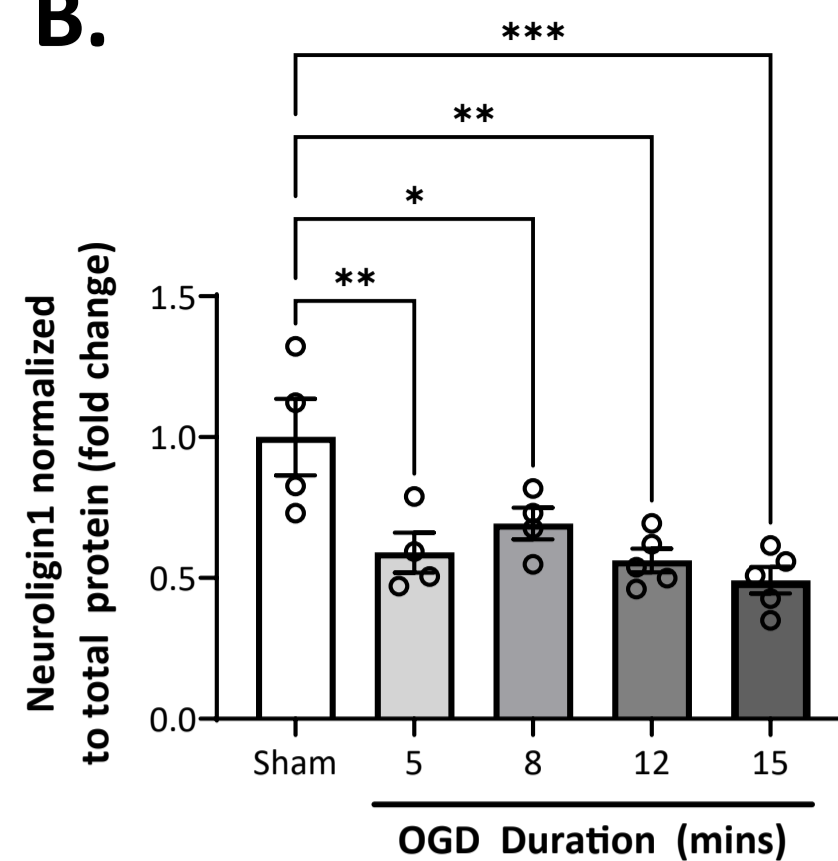**D.**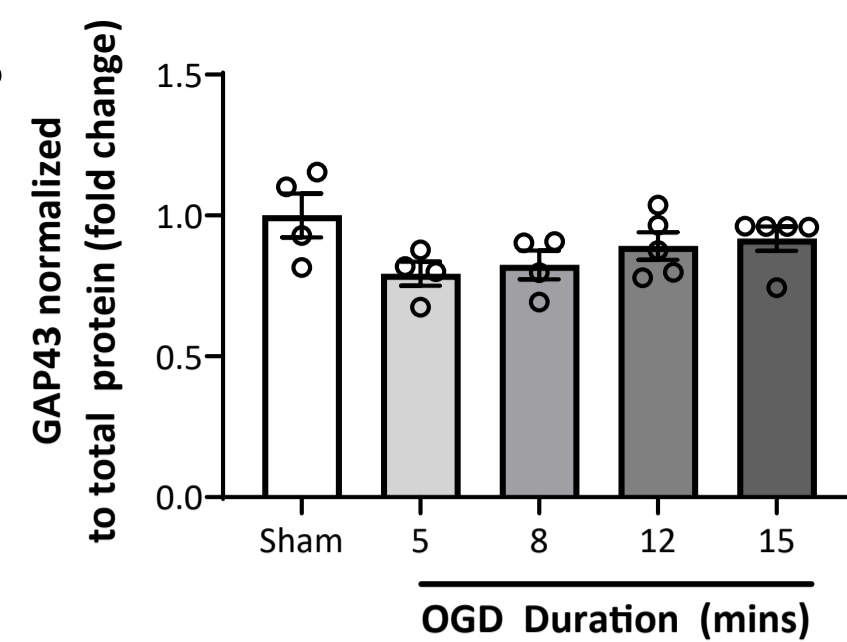**E.**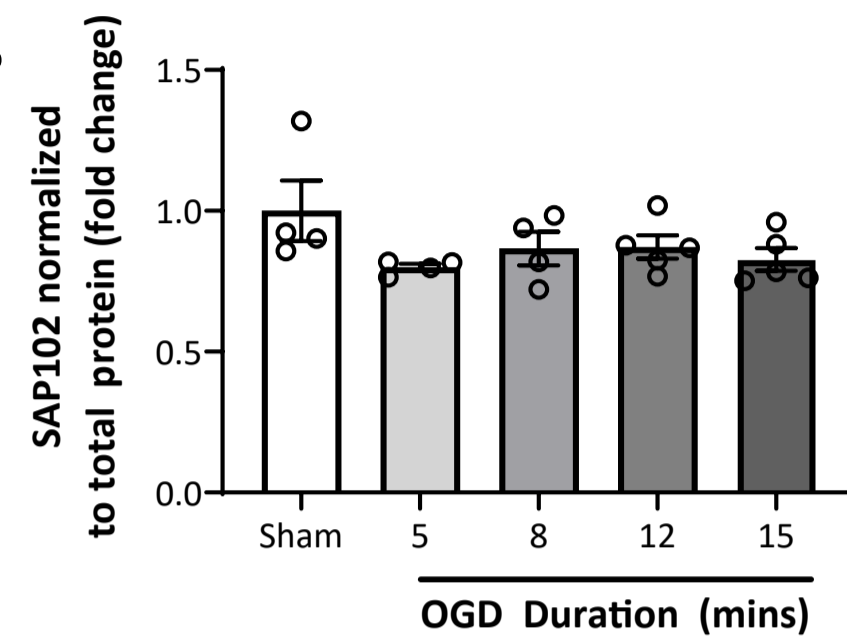**F.**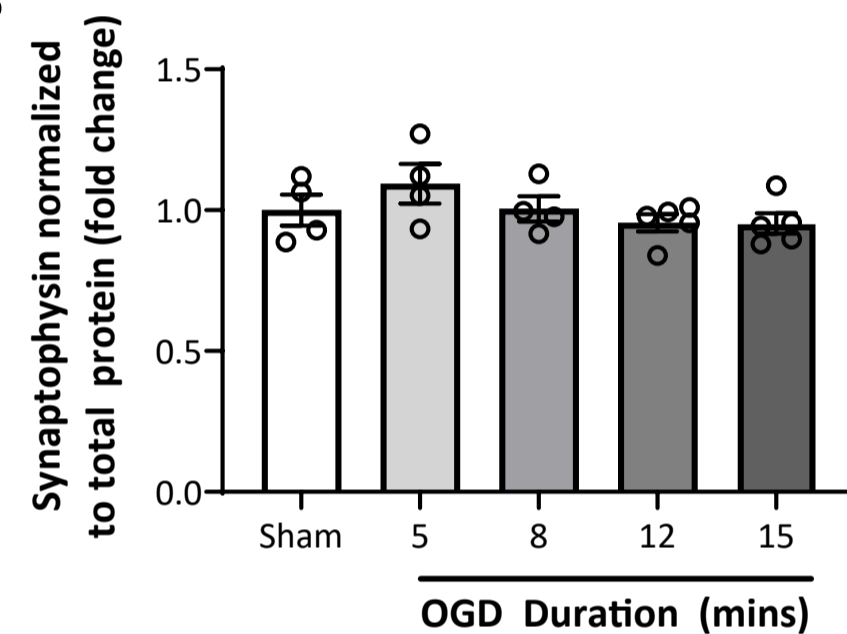**G.**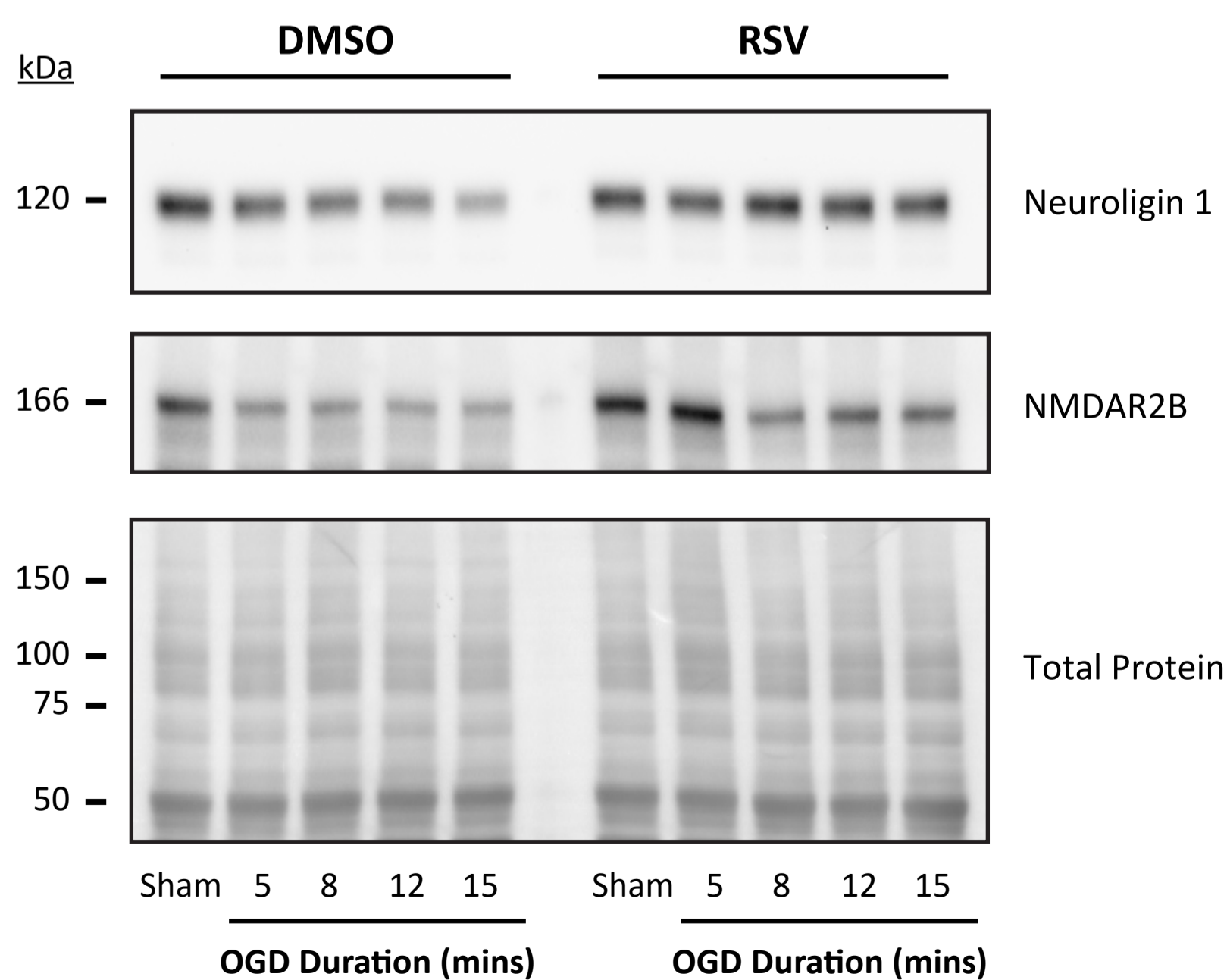**H.**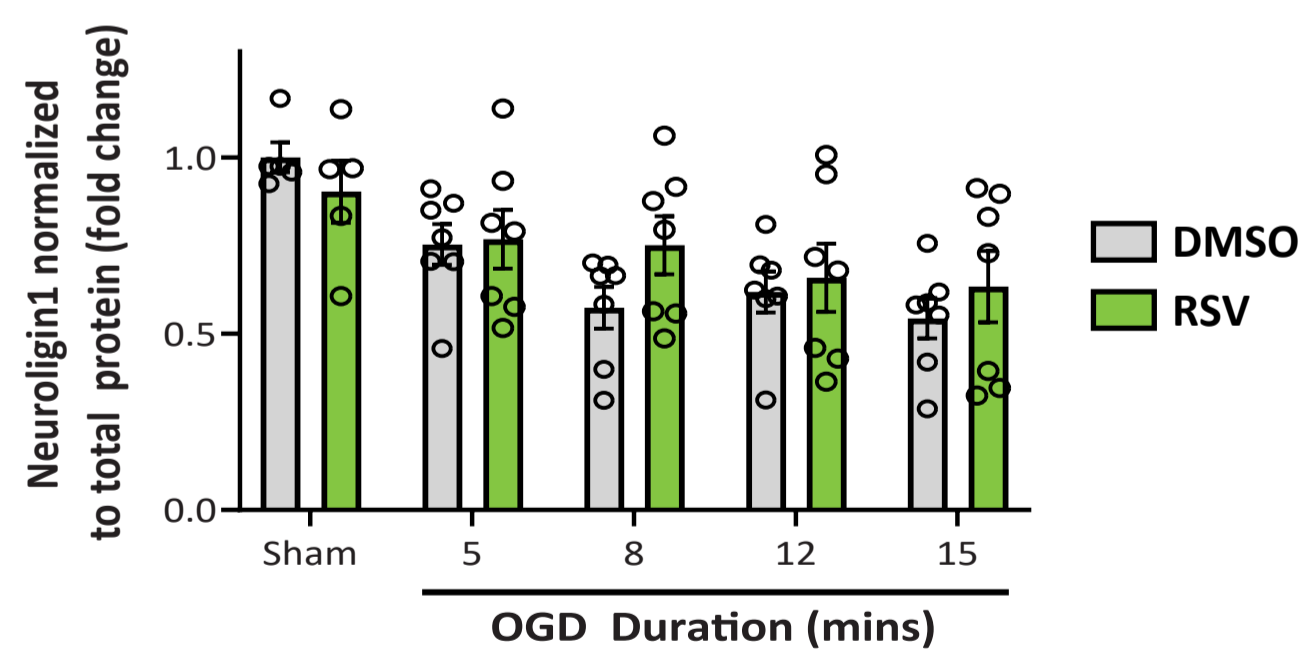**I.**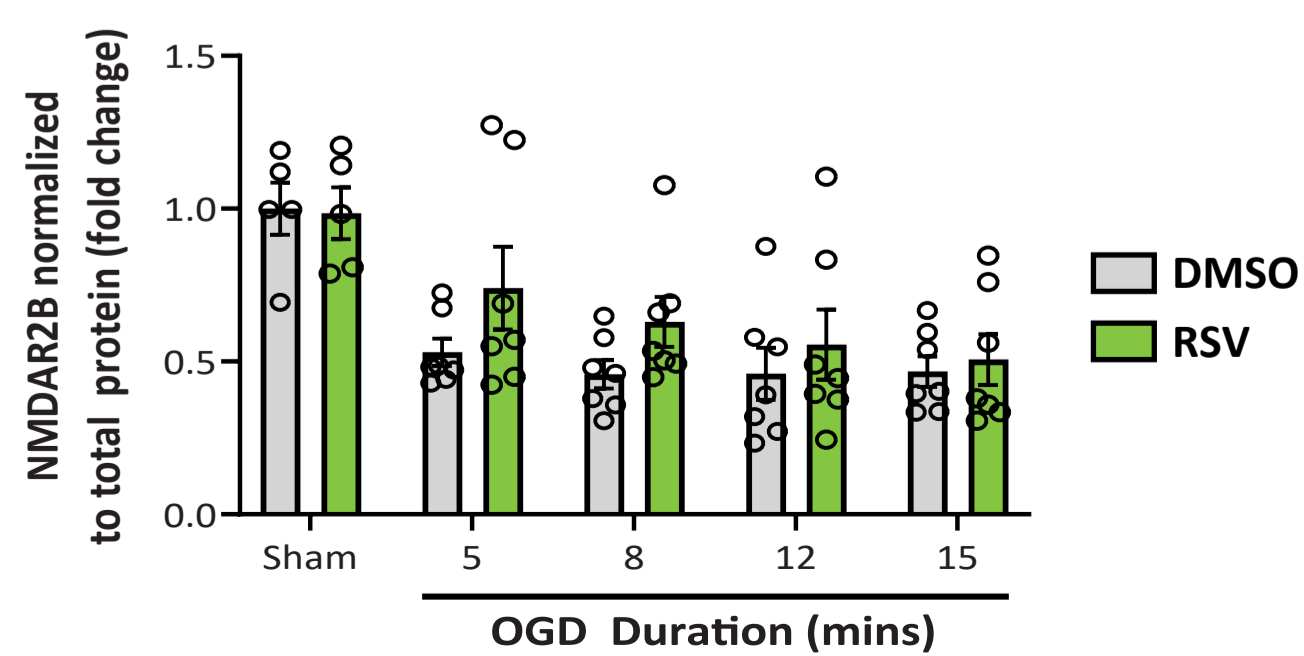

Supplement: Supplementary file 7 — Supplementary file7 (PDF 3121 kb) [file 13311_2023_1386_MOESM7_ESM.pdf]
