# Supplementary material for: Resveratrol Preconditioning Protects Against Ischemia-Induced Synaptic Dysfunction and Cofilin Hyperactivation in the Mouse Hippocampal Slice
Source: Neurotherapeutics. 2023 May 19;20(4):1177–97. doi: 10.1007/s13311-023-01386-0 (PMC10457274; doi:10.1007/s13311-023-01386-0)
Supplement: Supplementary file 6 — Supplementary file6 (PDF 325 kb) [file 13311_2023_1386_MOESM6_ESM.pdf]

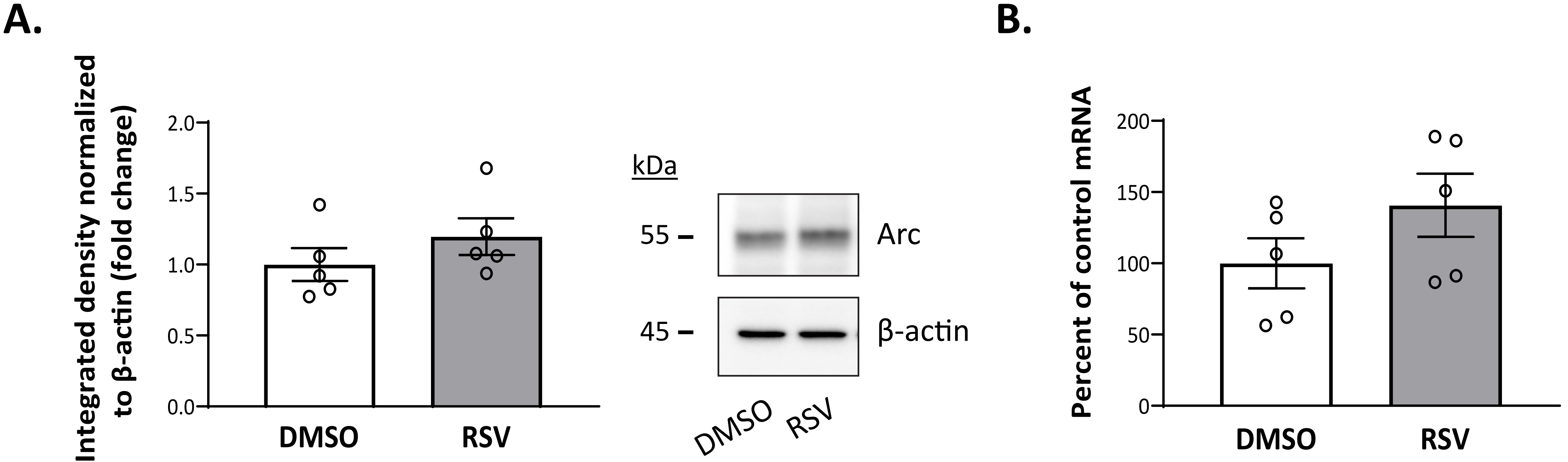
A.
Integrated density normalized to β-actin (fold change)
2.0
1.5
1.0
0.5
0.0
DMSO
RSV
kDa
55
45
Arc
β-actin
DMSO
RSV
B.
Percent of control mRNA
200
150
100
50
0
DMSO
RSV
